# Tracing tumorigenesis in a solid tumor model at single-cell resolution

**DOI:** 10.1038/s41467-020-14777-0

**Published:** 2020-02-20

**Authors:** Samantha D. Praktiknjo, Benedikt Obermayer, Qionghua Zhu, Liang Fang, Haiyue Liu, Hazel Quinn, Marlon Stoeckius, Christine Kocks, Walter Birchmeier, Nikolaus Rajewsky

**Affiliations:** 1 Systems Biology of Gene Regulatory Elements, Berlin Institute for Medical Systems Biology, Max Delbrück Center for Molecular Medicine in the Helmholtz Association, Berlin, Germany; 2 Core Unit Bioinformatics, Berlin Institute of Health, Charité – Universitätsmedizin Berlin, Berlin, Germany; 3 Signal Transduction in Development and Cancer, Max Delbrück Center for Molecular Medicine in the Helmholtz Association, Berlin, Germany; 4 New York Genome Center, New York, NY USA; 5 Present Address: Southern University of Science and Technology, Shenzhen, China

**Keywords:** Cancer stem cells, Computational biology and bioinformatics, Proteomics, Transcriptomics, Oncogenesis

## Abstract

Characterizing the complex composition of solid tumors is fundamental for understanding tumor initiation, progression and metastasis. While patient-derived samples provide valuable insight, they are heterogeneous on multiple molecular levels, and often originate from advanced tumor stages. Here, we use single-cell transcriptome and epitope profiling together with pathway and lineage analyses to study tumorigenesis from a developmental perspective in a mouse model of salivary gland squamous cell carcinoma. We provide a comprehensive cell atlas and characterize tumor-specific cells. We find that these cells are connected along a reproducible developmental trajectory: initiated in basal cells exhibiting an epithelial-to-mesenchymal transition signature, tumorigenesis proceeds through Wnt-differential cancer stem cell-like subpopulations before differentiating into luminal-like cells. Our work provides unbiased insights into tumor-specific cellular identities in a whole tissue environment, and emphasizes the power of using defined genetic model systems.

## Abstract (web summary)

Understanding tumour development at a granular level is a challenge in solid tumours. Here, the authors provide a cell atlas across tumour development in a genetic model of salivary gland squamous cell carcinoma using single-cell transcriptome and epitope profiling.

## Introduction

Solid tumors represent one of the main causes of morbidity and mortality worldwide. The molecular understanding of what drives carcinogenesis and tumor progression remains elusive though. This is in part due to the great extent of intra- and intertumoral heterogeneity in human tumors which confound a vast diversity of genetic and epigenetic factors that are subject to constant changes as a result of intrinsic and environmental cues^1^. Additionally, clinical samples are often derived from advanced or mixed tumor stages where information related to the initial induction of the cancer is frequently lost.

Another challenge consists in the limited availability of methods, which allow to disentangle the highly complex composition of the diseased tissues where cancer cells coexist together with tumor-associated and non-tumoral elements of the tumor microenvironment^2^. Advances in single-cell technologies have recently enabled several studies to elucidate the cellular complexity of a given tumor in more detail or to characterize specific tumor subtypes^3–8^. These findings support hierarchal models of tumor initiation by cancer stem cells (CSCs) that proliferate and differentiate, and induce heterogeneities in cancer cell phenotypes. CSCs are defined by their inherent ability to initiate and drive tumor growth and resistance to conventional treatment strategies^9^. Wnt signaling has been recognized to be a key driver for the initiation and maintenance of CSCs^10^, and it appears that stemness can be reversibly acquired and lost via epigenetic or environmental triggers, such as metabolic reprogramming or epithelial-to-mesenchymal transition (EMT)^11,12^. CSCs have thus become a prime focus for investigating molecular mechanisms that control tumorigenesis and metastasis. Their study, however, has been difficult due to their low frequency, unclear cell surface immunophenotype and other variable biological properties during disease progression^13^.

While emphasizing the great molecular diversity of human cancers, recent single-cell studies also revealed extensive coupling between different molecular levels. For instance, differences in genomic alteration patterns correlated with substantial transcriptional differences in head and neck cancer^7^. In contrast, in gliomas and breast cancer, the transcriptomes of different tumors were found to share similar differentiation or stemness signatures that were independent of genetic lineage relationships^3,6,14^. Moreover, complex relationships between tumor expression programs and tissue-of-origin transcriptomic signatures were found^8^, underscoring the need to comprehensively profile not only the tumor and its microenvironment but also histologically normal tumor-adjacent tissues as well as healthy controls in order to gain a full understanding of the complex feedback between tumor and host tissue^15^. Altogether, this accentuates the need to use controlled model systems to obtain a deeper understanding of general mechanisms that are intrinsic to tumorigenesis.

In a previous study, we found that high Wnt/β-catenin and low Bmp signaling were characteristic for aggressive forms of salivary gland and head and neck squamous cell carcinomas (SCCs) in humans^16^. With the aim to better understand these Wnt-specific mechanisms, we thus created a mouse model^16^ with *K14-cre*-driven *β-catenin* gain-of-function (β-cat^GOF^) and *Bmpr1a* loss-of-function (Bmpr1a^LOF^) mutations. We showed that these mice developed very specific salivary gland SCCs within 100 days after birth which contained highly self-renewing Wnt-dependent CD24^+^CD29^+^ CSCs which, upon isolation and injection into NOD/SCID mice, produced fast-growing tumors^16,17^. These CSCs showed high activity of the stem cell-associated SSEA1 marker as well as nuclear β-catenin and Wnt-specific target genes such as *Axin2* which were not found in other subpopulations within the tumor^16^.

**Fig. 1:**
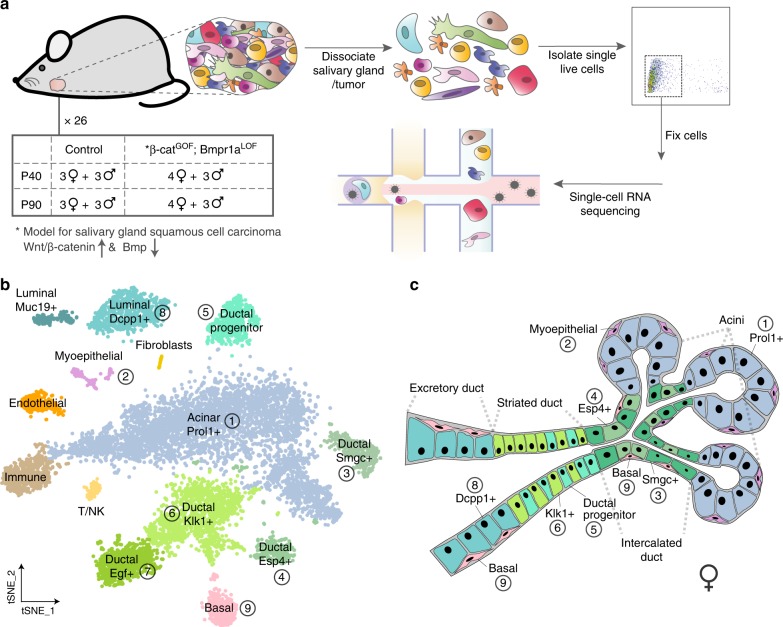
A comprehensive salivary gland cell atlas. **a** Experimental strategy to systematically dissect the cellular diversity in solid tumors. Submandibular salivary glands were individually dissected, dissociated and single, live cells isolated by FACS. Cells were immediately fixed in methanol and further processed to profile their transcriptomes by a high-throughput droplet-based single-cell approach. Each biological replicate corresponds to the cells of one submandibular gland from a control or tumor-bearing, female or male mouse at a defined stage as indicated. **b** tSNE representation of single-cell data from control salivary glands shows that cells cluster into 14 groups based on their transcriptome similarity. Clusters are colored and shaded according to the expression of both novel and known marker genes for epithelial and non-epithelial cell types. Turquoise—blue—green: luminal—acinar—ductal, pink: basal, purple: myoepithelial, shades of brown, yellow and orange: non-epithelial (immune, endothelial, fibroblasts, T/NK). **c** Anatomical sketch of the female submandibular gland based on single-cell transcriptome data, available literature (see text for references) and validations in tissue sections by immunofluorescence.

To gain a more basic understanding of tumorigenesis, we here used single-cell transcriptomics together with our Wnt-dependent double-mutant salivary gland SCC mouse model^16,17^ to systematically study CSCs in a controlled setting in vivo. Our setup (Fig. 1a) enabled us to build a high-resolution salivary gland cell atlas, to dissect tumor heterogeneity in a whole tissue environment and to identify CSC-like cells de novo directly from solid tumor samples. We show that tumor-specific epithelial cells consist of luminal- and basal-like cells as well as a small, but distinct CSC-like population. Further molecular characterization together with pathway and lineage analyses allowed us to infer and reconstruct a robust trajectory of the tumor progression. We found that upon activation of *β-catenin* gain- and *Bmpr1a* loss-of-function mutations in basal cells, tumorigenesis is initiated by expression of an EMT signature and proceeds through heterogeneous populations of CSC-like cells driven by differential Wnt signaling, before differentiating into luminal-like cells. Our work reveals several genes and expression patterns that may be fundamental in the regulation of tumorigenesis, and provides a novel and unbiased approach to study CSCs from a developmental perspective.

## Results

### Single-cell RNA sequencing of salivary gland tumors

To identify and characterize the cellular heterogeneity that is specific to the solid tumor context, we first established controlled ways to dissociate tumor-bearing (double-mutant: β-cat^GOF^; Bmpr1a^LOF^) and control salivary glands into high-quality single-cell suspensions (Fig. 1a). After dissociation, dead cells and enucleated cellular debris were excluded and live intact cells obtained by fluorescence-activated cell sorting (FACS) (Supplementary Fig. 1a). Cells were directly sorted into methanol for fixation^18^, and further processed to profile their transcriptomes by a high-throughput droplet-based approach (Drop-Seq)^19^. In total, 26 single-cell RNA libraries were generated from 12 control and 14 double-mutant (tumor-bearing) salivary glands of either female or male mice from an early and a late tumor stage at postnatal days 40 (P40) and 90 (P90), respectively (Fig. 1a). To validate our experimental approach, we compared all single-cell samples, computationally pooled by disease status (control or double-mutant), to bulk mRNA-seq data that were generated from equivalent, freshly dissected but unprocessed, salivary glands (Supplementary Fig 1b). Although gene expression levels correlated better within experimental procedures and samples grouped by genotype, correlations between all samples were generally high (*R* ≥ 0.74). Additionally, comparison of global transcript counts (Supplementary Fig. 1c) show that individual single-cell RNA libraries correlated well to each other (*R* ≥ 0.8) with no apparent bias towards the disease-, sex-, stage-related status or the experimental batch in which a specific sample was processed. After computational cell selection and filtering, we obtained a total of ~23,000 cells from 26 individual salivary glands (Supplementary Fig. 1d) and typically detected a median of ~500 genes and ~1000 unique molecular identifiers (UMIs) per cell (Supplementary Fig. 1e, f). To quantify sample-to-sample variation and the extent of possible batch effects, we developed an entropy-based approach^20^ to measure how evenly cells’ nearest neighbors are distributed among different samples. This analysis showed that although these distributions were not completely random, ~80% of the variance was explained by the three major biological variables (genotype, sex, and stage) (Supplementary Fig. 2a). Further, cell distributions were largely balanced except in cell types that are affected by one of these biological variables (Supplementary Fig. 2b). We thus conclude that our protocol is reproducible given biologically-relevant sample differences.

### A comprehensive salivary gland cell atlas

In order to provide a comprehensive cell atlas of the salivary gland that could also serve as an appropriate reference to the tumor context, we first pooled and analyzed single-cell datasets from control salivary glands using Seurat^21^ (Fig. 1b). After inspection of marker genes, we assigned the cell type identity to clusters, for which the expression of specific genes has been reported in previous literature^22–31^, and molecularly characterized and validated additional cell types for which no or only ambiguous information was available (Fig. 1b, c; Supplementary Figs. 3, 4). In agreement with previous studies^32–34^, we observed that *Egf* and *Smgc* were expressed in cell populations with strongly sex-dependent representation (Supplementary Fig. 3) and identified several other marker genes with similar patterns (Supplementary Fig. 4a). Interestingly, we also noted that Dcpp1+ cells were more abundant in tissues from mice at the P40 than at the P90 stage and validated this finding by immunofluorescence in tissue sections (Supplementary Fig. 3b, c). Altogether, this indicates that we can reliably identify and characterize cell types at high resolution and provide evidence that the ductal composition of healthy salivary glands is sexually dimorphic and stage-dependent. Our approach combined with imaging-based validations enabled us to chart all epithelial cells onto a consolidated anatomical sketch depicting female- (Fig. 1c) and male-specific (Supplementary Fig. 3e) features.

### Identification of cancer stem cells and other tumor-specific cell populations

**Fig. 2:**
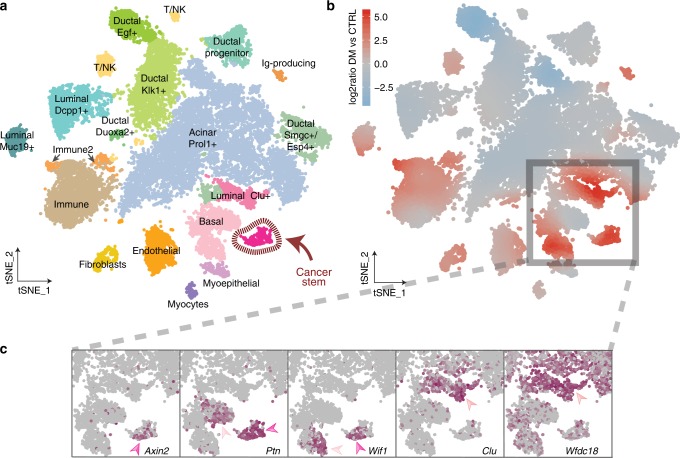
Single-cell sequencing identifies a distinct cancer stem cell-like and other tumor-specific cell populations. **a** Clustering of combined single-cell sequencing data from control and double-mutant (β-cat^GOF^; Bmpr1a^LOF^) tumor-bearing salivary glands shown in the tSNE. Tumor-specific epithelial cell clusters are shown in the lower right part of the tSNE in shades of pink: cancer stem, basal, luminal Clu+ cells. **b** Same tSNE plot showing the relative local densities of control and double-mutant cells. Color gradients reveal clusters that are predominantly represented by double-mutant (red), control (blue) or both double-mutant and control cells (gray). **c** Expression of *Axin2, Ptn, Wif1, Clu*, and *Wfdc18* in the tumor-specific epithelial subset of the tSNE representation as indicated by a color scale ranging from gray (no expression) to maroon (high expression).

To systematically uncover cells that were specific to the tumor samples, we analyzed and clustered cells pooled from all single-cell datasets of control and double-mutant samples (Fig. 2a). This combined analysis enabled us to recapitulate all previously identified cell types without the need to resort to advanced sample alignment methods^21^ as cells from both control and double-mutant mice were distributed evenly on the tSNE for many clusters as shown in the local density plot (Fig. 2b). However, in line with our entropy-based analysis (Supplementary Fig. 2), we noted that a number of cell types, including stromal and immune-related cells were significantly more abundant in double-mutant (Supplementary Fig. 5a, Supplementary Fig. 6), while acinar and some ductal cell types were accordingly more prevalent in control tissues (Supplementary Fig. 5b).

In particular, our analysis revealed clusters of epithelial cells with transcriptional profiles unique for the tumor context. This included luminal- and basal-like cells as well as a small but distinct cancer stem cell (CSC)-like population in which Wnt-specific genes were activated (Fig. 2b, c). Among others, the expression of several genes such as *Axin2*, *Ptn*, *Wif1*, *Clu* and *Wfdc18* was particularly characteristic for these tumor-specific cell clusters (Fig. 2c). Using these genes as markers, we further confirmed that the tumor-specific epithelial cells identified in our data were truly located in tumor regions. Immunofluorescence analysis of submandibular gland tissue sections from double-mutant mice showed that antibodies against these gene products positively stained cells within evident tumor lesions (Supplementary Fig. 7). To further increase tumor-specific resolution, we included other epithelial markers where possible. In line with our transcriptome data, Clu and Wfdc18 distinctively stained K8-positive luminal-like cells within the tumor region. Nuclear β-catenin is considered to be the hallmark of active Wnt signaling which ultimately drives the expression of its target genes^35,36^ and was previously described to be a key feature of CSCs in several cancers including our model^16,17,37^. We, therefore, used this as an additional marker, and found that nuclear β-catenin-positive cells were generally K8 negative and greatly overlapped with high Axin2, but only partially with Ptn and Wif1 stainings. Taken together, we identified a small subset of tumor-specific epithelial cells and provide a marker set that can identify them both at the RNA and protein level.

### Simultaneous quantification of mRNA and cell surface proteins resolves immune cell diversity

**Fig. 3:**
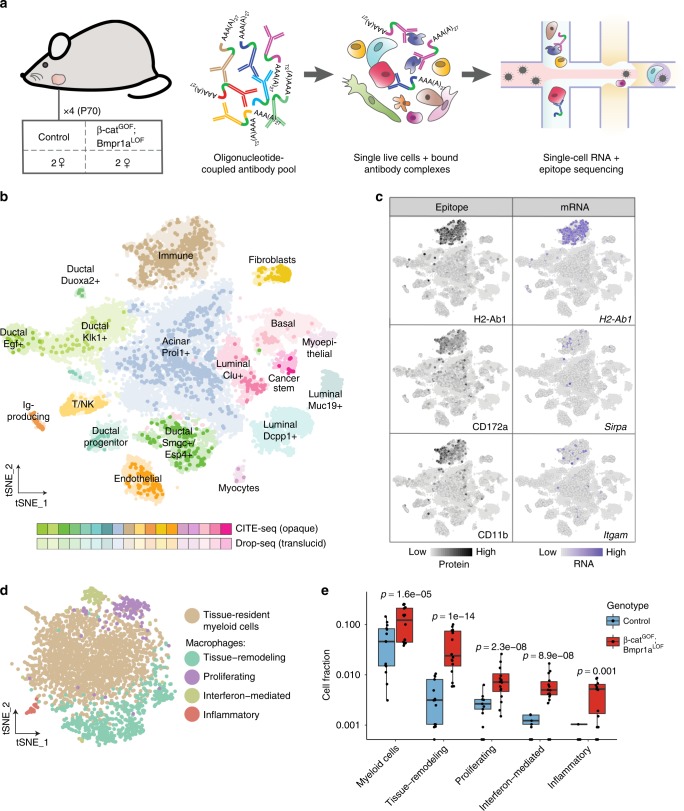
Combined mRNA and epitope profiling from single cells resolves immune cell diversity in the tumor microenvironment. **a** Overview of cellular indexing of transcriptomes and epitopes by sequencing (CITE-seq) experiments (*n* = 4). Single live cells were prepared as described earlier. A pool of 63 oligonucleotide-coupled antibodies was incubated with the cell suspension from one submandibular gland, washed and further processed by Drop-seq. The oligonucleotides contain an antibody-specific barcode, a PCR handle and are polyadenylated for capture by the Drop-seq primer beads. **b** mRNA-based clustering of all single-cell datasets. Cells from the CITE-seq experiments are highlighted (opaque colors) over those from previous ‘Drop-seq only’ transcriptome datasets (translucid colors). **c** Epitope and mRNA signals in cells from CITE-seq experiments for selected immune-specific markers. **d** Subclustering of the immune cluster (shown in **b**) identifies 4 macrophage subpopulations. **e** Contributions of cells from control or double-mutant samples to immune subpopulations. *P*-values from mixed effects binomial model using 3207 cells in 30 samples. Boxes span the 25th to the 75th percentile, whiskers 1.5 times the interquartile range.

Unbiased transcriptional profiling identified four clusters of various adaptive and innate immune cells. T or NK cells (‘T/NK’; *Ccl5, Nkg7, Cd7*)^38^ and tissue-resident monocytic phagocytes (‘immune’) were already present in control salivary glands (Fig. 1b). In the tumor context, ‘immune’ cells were significantly more abundant in double-mutant compared to control samples (Supplementary Fig. 5a). Additionally, new populations of monocytic cells (‘immune2’) and terminally differentiated B plasma cells (‘Ig-producing’; *Ly6c2, Slpi, Xbp1*)^39^ emerged in double-mutant tissues showing an influx into or activation of immune cells in the tumor environment (Fig. 2a; Supplementary Fig. 5a). To investigate this tumor-specific immune compartment in more detail, we used ‘CITE-seq’^40^ in conjunction with a panel of 63 oligonucleotide-coupled antibodies mainly directed against immune and some epithelial cell surface proteins (see Supplementary Table 1). This allowed us to simultaneously quantify mRNA transcripts and protein epitopes from single cells of freshly dissociated ~P70 control and tumor-bearing salivary glands (Fig. 3a). Implementation of CITE-seq did not introduce any obvious transcriptional biases as mRNA-based clustering with our earlier obtained transcriptome datasets recapitulated all cell clusters with cells from the CITE-seq experiments evenly distributed in the tSNE (Fig. 3b). Integration of cell surface protein information enhanced the signals for specific markers such as CD172a and CD11b (Fig. 3c), confirming that ‘immune’ and ‘immune2’ clusters predominantly consist of myeloid cells from the monocytic lineage. Subclustering of the combined ‘immune’ clusters revealed four cell subsets that exhibited signatures of tumor-associated macrophages (TAMs) with diverse activation and functional states (Fig. 3d, e). These TAMs differed in expression of genes associated with tissue-remodeling (*Vim, Mmp12, Fn1*)^41–43^, proliferating (*Hmgb2, Mki67*)^44,45^, interferon-responding (*Isg15, Rsad2, Irf7*)^46,47^ or inflammatory (*Irg1, S100a9, S100a8*)^48–50^ properties (Supplementary Fig. 8). In summary, our results suggest that the immune landscape in this Wnt-dependent salivary gland tumor model is dominated by myeloid cells and TAMs, accompanied by a small population of tumor-associated B plasma cells. Consistent with the aggressive nature of these tumors, infiltration by inflammatory T cells seemed low^51^ with T/NK mostly corresponding to tissue-resident cells. Our data support the newly emerging concept that TAMs exhibit a wide and continuous spectrum of functional and differentiation states^20,52^ rather than conforming to a defined polarizing tumor-supporting vs. tumor-suppressing model.

### Subclustering reveals two subpopulations of cancer stem and basal cells

**Fig. 4:**
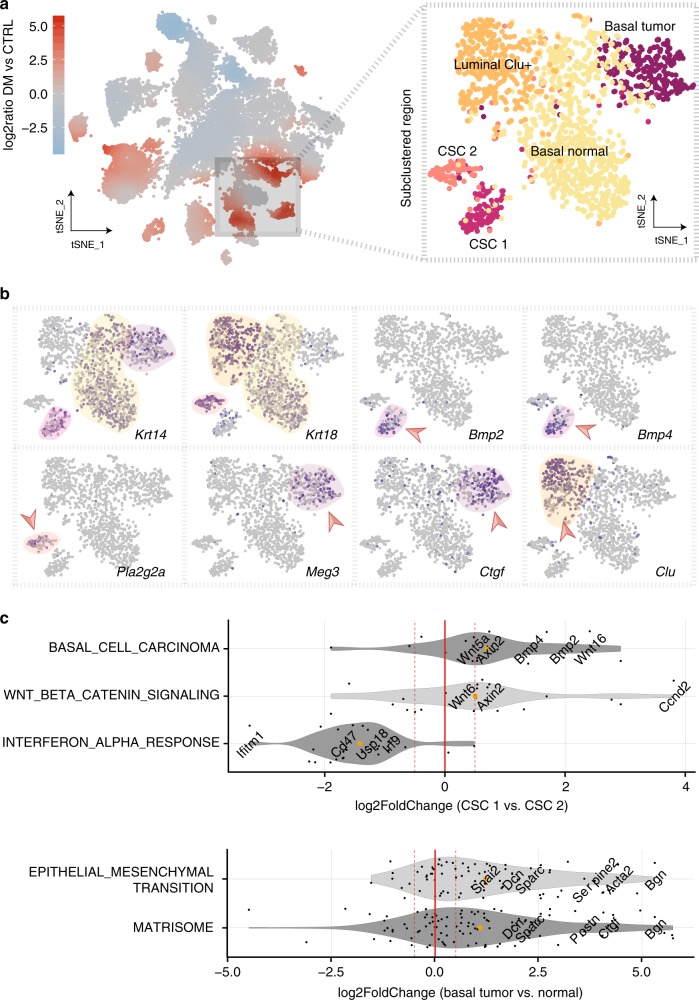
Subclustering reveals additional heterogeneity in cancer stem cell-like and basal cells. **a** Subclustering of the tumor-specific epithelial subset identifies two cancer stem cell-like (‘CSC 1’ and ‘CSC 2’) and two basal cell (‘basal normal’ and ‘basal tumor’) subpopulations. **b** Expression of several genes in the subclustered tumor-specific tSNE representation as indicated by a color scale ranging from gray (no expression) to dark blue (high expression) with regions of high expression highlighted. **c** Gene set enrichment analysis on differentially expressed genes between CSC-like or basal subclusters, respectively. Shown are log2 fold changes for all genes per pathway expressed in at least 5% of cells, with selected genes highlighted. Orange dots indicate the pathway mean, dashed lines the cutoff set on the pathway mean.

Since our data indicated heterogeneity within tumor-specific cells (Fig. 2b, c), we selected basal cells and CSC-like cells for subclustering (Fig. 4a), which showed that each of the two clusters consists of two distinct subpopulations. Inspection of the top marker genes (Fig. 4b; Supplementary Fig. 9a) revealed that the CSC 1 subpopulation exhibited more basal-like (*K14 *+ /*K5*+) Wnt-high (*Bmp2*, *Bmp4*, *Dkk4*+) whereas CSC 2 rather luminal-like (*K18*+) features. Moreover, we were able to distinguish the tumor-specific basal cell subset (‘basal tumor’) from the ‘normal’ one (‘basal normal’), as illustrated by projecting the subclustered cell populations back onto the original tSNE coordinates from the clustering of all samples (Supplementary Fig. 9c).

To functionally characterize these differences, we performed differential expression and subsequent pathway analyses between the respective pairs of CSC-like and basal subpopulations (Fig. 4c). This confirmed our initial observation in CSC-like cells as terms related to Wnt/β-catenin signaling and basal cell carcinoma were most significantly enriched in the ‘CSC 1’ compared to the ‘CSC 2’ subcluster. When comparing the two basal subpopulations, we found that the main systematic differences were linked to extracellular matrix (ECM) proteins or epithelial-to-mesenchymal transition (EMT) signatures, which were strongly upregulated in the tumor-specific basal subset. Specifically, we observed a more than two-fold upregulation of the EMT master regulator *Snai2* and strong induction of other characteristic genes such as *Serpine2*, *Sparc*, *Acta2*, *S100a6* or the TGF-β modulator *Bgn*^53^ (Fig. 4c, Supplementary Fig. 9, Supplementary Data 1, Supplementary Fig. 10), while many other canonical EMT markers such as *Zeb1/2*, *Twist1/2*, *Fn1* or *Cdh2* were insufficiently detected in our data to be tested for differential expression. To make our data easily accessible, we created a resource, which allows to access and interrogate our single-cell data for any gene of interest interactively via a web-based online tool (https://shiny.mdc-berlin.de/sc_msga/).

### Computational lineage analyses reconstruct tumorigenesis

**Fig. 5:**
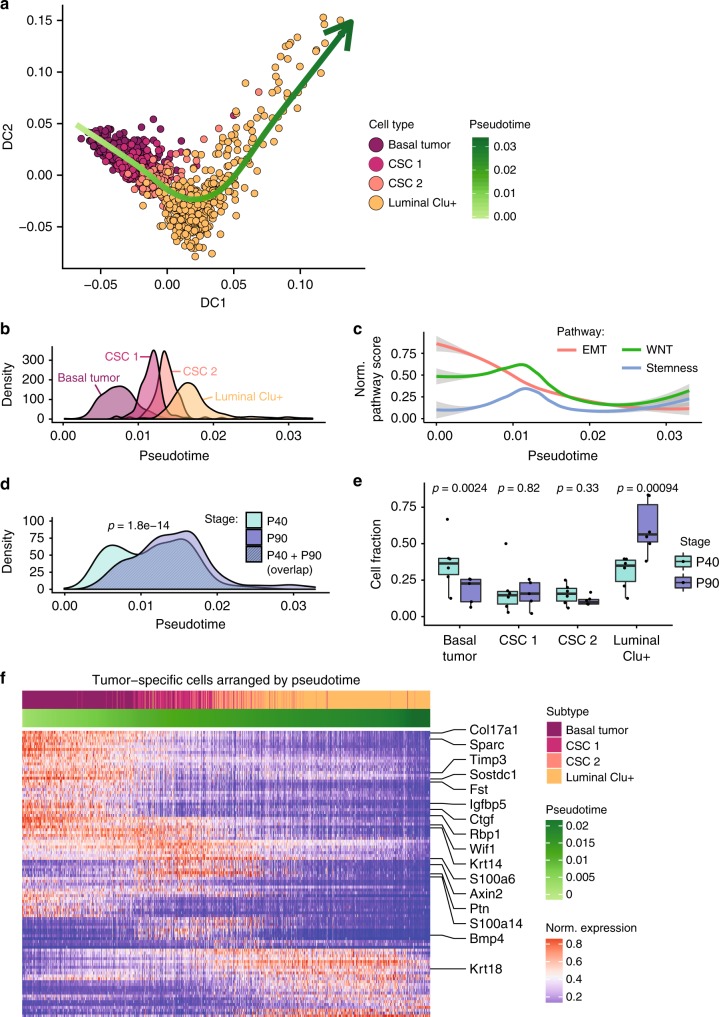
Computational lineage modeling allows to infer a robust trajectory of tumorigenesis. **a** Diffusion map of tumor-specific epithelial cell populations together with inferred trajectory obtained by smoothing diffusion coordinates over pseudotime. **b** Density plot of cells from the different subpopulations along pseudotime. **c** Normalized EMT, Wnt and stemness^64^ pathway scores, smoothed over pseudotime using LOESS regression (gray shading indicates 95% confidence intervals). **d** Density plot of cells from double-mutant P40 and P90 samples with ≥5 cells in the 4 subpopulations along pseudotime. *P*-value from linear model using 855 cells in 12 samples. **e** Proportions of the subpopulations from the same samples according to stage (*P*-values from binomial mixed effects model). Boxes span the 25th to 75th percentile, whiskers 1.5 times the interquartile range. **f** Heatmap of SAVER-imputed gene expression for the top 100 differential genes between the 4 subpopulations (top 16 are highlighted).

We further investigated to what extent tumor-specific cells were connected to one another and contributed to the tumorigenesis in our genetic model. We chose a diffusion map approach, which embeds data in low-dimensional space where distances between cells represent a gradual but stochastic continuation such as during developmental processes^54^. Together with pseudotemporal ordering, this analysis allowed us to predict a differentiation trajectory for tumorigenesis (Fig. 5a, Supplementary Fig 11a). In agreement with our model, in which tumors are induced by activation of *β-catenin* and *Bmpr1a* mutations via the basal-specific *K14-cre* promoter^16,17^, we find that this trajectory initiates in basal tumor cells, further proceeds through the two CSC-like subpopulations, and ends in the luminal Clu+ cell cluster with continuous transitions in between (Fig. 5a, b). We show that this trajectory is robust and reproducible across individual double-mutant tumor samples (Supplementary Fig. 11b) and that removal of CSC-like cells from the analysis still predicts the tumorigenesis path although disrupting cell connectivity (Supplementary Fig. 11c).

To validate this trajectory, we made use of the different time points (P40 and P90) at which we collected samples and monitored the contributions of tumor-specific cells pooled by stage (Fig. 5d, e; Supplementary Fig. 12) instead of cell type. In agreement with our model, we found that the number of cells from the early P40 tumor stage was significantly increased at the initial phase of tumorigenesis compared to that from the late P90 one, while the reverse was true at a more advanced phase. Stage-specific quantification of tumor-specific cells showed that although relative proportions of both CSC-like populations were similar, basal tumor and luminal Clu + cells were, in fact, significantly more and significantly less abundant in P40 cells than in P90 cells, respectively (Fig. 5e).

To study potential changes in global expression dynamics along the trajectory, we took the most differentially expressed genes between the four subpopulations and ordered their expression by pseudotime (Fig. 5f, Supplementary Fig 13). The results replicated the differential expression analyses summarized in Fig. 4c, and showed that we could additionally capture more subtle differences for individual genes for which the expression changed as a function of the tumor progression. We found that EMT-related genes such as *Ctgf*^55^ and *Sparc*^56,57^ were first activated in basal tumor cells, and that their expression extended and decreased within the cell population (*Ctgf*) itself or throughout both CSC-like subpopulations (*Sparc*) as tumorigenesis proceeded (Fig. 5f, Supplementary Fig. 13). A similar pattern was observed for *Meg3*, a long non-coding RNA previously found to regulate EMT^58^ as well as the Wnt/β-catenin and p53 pathways^59,60^.

While several genes (e.g., *Ptn*, *S100a6*, *S100a14*) were expressed in a more ubiquitous manner in CSC-like cells, Wnt target genes such as *Axin2* and *Bmp4* were specifically switched on in the CSC 1 subpopulation and continued to be only mildly expressed and subsequently inactivated in CSC 2 (Supplementary Fig. 13). However, in the latter, increased expression of the transcriptional regulators *Nupr1* and *Elf3* could be detected, both genes with known functions in epithelial cell differentiation and tumorigenesis^61,62^. Finally, *Clu*, and *Wfdc18*, which we identified as being specific to luminal Clu+ cells (Fig. 2), were expressed at a later stage towards the end of our inferred trajectory. Validating previously proposed associations between EMT, Wnt signaling and stemness^63^, we found that gradual loss of the EMT expression signature correlated with activation of Wnt signaling and induction of known CSC marker genes in head and neck squamous cell carcinoma^64^ (Fig. 5c). Moreover, by inspecting differential pathways with mean log2 fold changes below the cutoff imposed in Fig. 4c, we identified dysregulated metabolic signatures suggesting that activation of the EMT program in basal tumor cells is accompanied by metabolic reprogramming from oxidative phosphorylation towards glycolysis (Supplementary Fig. 14). Together, our results show that our approach can identify specific genes and expression patterns potentially regulating and driving tumorigenesis.

## Discussion

In this study, we created a high-resolution salivary gland cell atlas and systematically dissected the cellular heterogeneity in a genetically-controlled, Wnt-dependent mouse model of a solid tumor. We identified, molecularly characterized and validated cell clusters that were specific to the tumor and established their lineage relationship by uncovering the progenitor and progeny populations of CSCs.

Different from other studies involving single-cell transcriptome profiling of human biopsy samples^5–7^, we could not computationally pinpoint the tumor cells by global copy-number variation (not present in our data), nor by identifying *β-catenin* and *Bmpr1a* mutations (not detectable) or EYFP-positive cells (mRNA transcripts not sufficiently captured by our sequencing method). Instead, we leveraged the reproducibility and large sample size of our data to systematically compare cells from control and tumor-bearing tissues to reliably and robustly identify those that were specific to the tumor samples, and confirmed these results by immunostainings in tissue sections.

Although only representing <1% of cells in our data, we were able to detect and extensively characterize CSC-like cells without relying on pre-defined surface markers, which often also target unrelated cells^9,13^. We found that Wnt target genes such as *Axin2* and *Bmp4* were exclusively activated in this small population, but also other genes with highly tumor-specific functions such as *Ptn* (promoting tumor angiogenesis^65^) and *S100a14* (involved in tumorigenesis^66^). Further investigation revealed that these CSC-like cells consist of a Wnt-high/K14+  and a luminal-like/K8+ subpopulation, also characterized by differential expression of the transcriptional regulators Nupr1 and Elf3, which are thought to promote metastasis and the induction of chemoresistance^61,62^. Further experiments will be required to functionally characterize these CSC subpopulations and to address their stemness potential. Nevertheless, it has been recognized that different stem cell types with tissue renewal capacity can reside within the same tissue, that a continuum of stem cell states may provide a higher degree of flexibility^67^, and that Wnt signaling is essential for stem and progenitor cell formation and function throughout development^10,68^. Our results indicate that the stabilizing β-catenin mutations in our mouse salivary gland SCC model trigger similar processes, as uncontrolled Wnt signaling can lead to aberrant expansion of stem cells or confer stem cell behavior, paving the way for malignant proliferation^9^.

Since the tumor in our model was driven by a K14-cre, we argued that the CSCs most likely originated from the tumor-specific basal population, characterized by an EMT expression signature. The importance of the EMT program is well established as a major mechanism for the ‘invasion-metastasis cascade’ in cancer biology^63^. With an ability to degrade and reorganize the ECM, it promotes the loss of cell-cell adhesion and the acquisition of migratory and invasive traits. In fact, EMT signatures have been observed in the tumor microenvironment^69,70^, but also in malignant cells^7^, and its critical role for the induction of a stem-like phenotype has only been recognized over the last years^12^. It has emerged that epigenetic and environmental cues can control EMT without introducing new genetic alterations, that this transition is therefore reversible, rarely fully executed under physiological or malignant conditions, and that an intermediate state between the poles of fully epithelial and mesenchymal cell identity is particularly favorable for the induction of the CSC phenotype^63,71^. Specifically, Snai2/Slug in coorporation with Sox9 have been identified as stemness- and EMT-inducing factors in the basal layer of the mammary epithelium^72^. Moreover, the activity of these transcription factors can promote the existence of transient epithelial cell populations which can convert to stem cells with long-term tissue reconstituting ability^72^, and we propose that similar mechanisms play an essential role in our system.

Further, while connections between Wnt signaling both downstream and upstream of the EMT have long been drawn^63^, we find here that genetic Wnt activation and Bmp deactivation in our model triggers EMT (presumably through Snai2) and metabolic reprogramming, before other Wnt targets, such as *Bmp2*/*4* or *Dkk2*/*4* can be detected.

Our data allowed us to put the different tumor-specific cell populations on a robust trajectory and to map the continuous shifts in cellular identities during tumorigenesis by means of diffusion maps, which have been widely used together with single-cell data to study differentiation dynamics during development^73,74^. We note, however, that the directionality of the inferred trajectory is not conclusively fixed and requires further investigation. Nevertheless, based upon the K14-cre-dependence of mutations triggering the tumor in our model as well as the relative timing of early- and late-staged samples, we hypothesized that the tumorigenesis here is initiated in basal cells. This model further suggests that after EMT induction, Wnt signaling was upregulated in one CSC subpopulation in concert with the acquisition of a stemness phenotype that persisted throughout another CSC subpopulation already primed towards a luminal-like cell fate, towards which tumorigenesis finally transitioned. Comparable to epithelial differentiation processes, we noticed a general K14+ to K8+ gradient indicating that tumor-specific cells shifted from exhibiting basal-like to more luminal-like characteristics along the trajectory. This was accompanied by a gradual loss of EMT markers and therefore also suggests a reversal to an epithelial cell identity. Moreover, we found that the luminal-like cells exhibited high expression of *Wfdc18*, a gene that we identified to be specific to the excretory and intercalated ducts in control tissues. This suggests that in our model, Wfdc18-positive cells in the tumor may have undergone a similar differentiation path as normal cells in these ductal compartments.

Our work demonstrates the importance of using controlled models to robustly and reproducibly study essential mechanisms of carcinogenesis and tumor progression which would otherwise not be possible. Finally, our approach provides a blueprint to molecularly identify markers and characterize transcriptional events that are fundamental in the regulation of tumorigenesis, and ultimately facilitate further clinical studies in the design of appropriate treatment strategies.

## Methods

### Mouse strains

K14Cre(Δneo), β-catenin^flox^, Bmpr1a^flox^ alleles, and Cre-inducible R26^EYFP^ reporter mice have been described, and mutant mice were analyzed for genotype and recombination by PCR^16,75–79^. To obtain the double mutants, homozygous mice carrying the β-catenin^flox^ gain-of-function, the Bmpr1a^flox^ loss-of-function allele and R26^EYFP^ were crossed with K14-Cre mice that were homozygous for the Bmpr1a^flox^ allele. Control mice were obtained by crossing K14-Cre mice with mice carrying the Cre-inducible R26^EYFP^ reporter. All mice used in this study had a C57BL/6 background. Animal experiments were approved by LAGeSo Berlin and performed according to EU and national institutional regulations.

### Tissue dissociation and single-cell sample preparation

Submandibular glands and primary tumor samples were collected, minced and dissociated with a GentleMacs Dissociator (Miltenyi Biotec) in digestion buffer (DMEM/F12 1:1 (Invitrogen), 1.67 mg/ml collagenase (Invitrogen), 1.33 mg/ml hyaluronidase (Sigma) and 1.67 mg/ml dispase (Invitrogen)). Cell suspensions were passed through a stainless filter (70 μm) and centrifuged at 900 × *g* for 5 min at 4 °C. Pellets were suspended in 10 ml Dulbecco’s modified Eagle/F12 1:1 medium and washed three times with PBS containing 10% fetal bovine serum (Invitrogen). Prior to sorting, cells were stained with DAPI, and then filtered through a 40 μm mesh. The FACS Aria (BD Biosciences) instrument was used for sorting and dead cells excluded by elimination of DAPI-positive cells and gates set to exclude cell clusters. Cells were directly sorted and fixed in ice-cold 80% methanol and stored at −80 °C until further processing.

### Drop-seq procedure, single-cell and bulk library generation, and sequencing

Monodisperse droplets of about 1 nl in size were generated using microfluidic PDMS devices (Drop-SEQ chips, FlowJEM, Toronto, Canada; pre-coated with Aquapel). Barcoded microparticles (Barcoded Beads SeqB; ChemGenes Corp., Wilmington, MA, USA) were prepared and flowed in using a self-built Drop-seq set up^19^ (Online-Dropseq-Protocol-v.3.1: http://mccarrolllab.com/dropseq/) as previously described^18^. Cell preparations and reagents were kept on ice and handled in the cold. Methanol-fixed cells^18^ were centrifuged at 3000–5000 × *g* for 5 min, rehydrated in 1 ml PBS + 0.01% BSA supplemented with RNAse inhibitors (1 unit/μl RiboLock, ThermoFisher), pelleted and resuspended again in 0.5 ml PBS + 0.01% BSA in the presence of RNAse inhibitors. Cells were manually counted by means of a hemocytometer and diluted to a suspension of typically ~50–100 cells/μl in PBS + 0.01% BSA. Droplets were collected in 50 ml Falcon tubes for ~13 min, corresponding to ~1 ml of combined aqueous flow volume (1  ml cells and 1 ml of beads). Droplets were broken immediately after collection and barcoded beads with captured transcriptomes were reverse transcribed and exonuclease-treated. First strand cDNA was amplified by equally distributing beads from one run to 24 PCR reactions (50 μl volume; 4 + 9 to 11 cycles). 20 μl fractions of each PCR reaction were pooled (total = 480 μl), then double-purified with 0.6x volumes of AMPure XP beads (Beckman Coulter). Amplified cDNA libraries were assessed and quantified on a BioAnalyzer High Sensitivity Chip (Agilent) and the Qubit dsDNA HS Assay system (ThermoFisher). If necessary, more cDNA was purified from the PCR reactions. 600 pg of each cDNA library was fragmented, amplified (12 cycles) and indexed for sequencing with the Nextera XT v2 DNA sample preparation kit (Illumina) using custom primers enabling 3’-targeted amplification as described^19^. The libraries were double-purified with AMPure XP Beads (0.6 × , 1 × ), quantified and sequenced on Illumina NextSeq500 sequencers (library concentration 1.8 pM; NextSeq 500/550 High Output v2 kit (75 cycles) in paired-end mode; read 1 = 20 bp using the custom primer Read1CustSeqB^19^, read 2 = 64 bp).

For bulk sequencing, RNA was extracted with Trizol (Invitrogen) from freshly dissected submandibular glands. RNA integrity was assessed on a BioAnalyzer RNA Nano Chip (Agilent). Strand-specific cDNA libraries were generated from 500 ng total RNA according to the Illumina TruSeq protocol (TruSeq Stranded mRNA LT Sample Prep Kit, Illumina). Libraries were sequenced on an Illumina NextSeq 500 sequencer using the High Output v2 Kit (150 cycles), single read: 150 bp, index read: 6 bp.

### CITE-seq experiments

Antibodies were covalently and irreversibly conjugated to DNA-barcoding oligonucleotides by iEDDA click chemistry as previously described^80^. The antibody panel (see Supplementary Table 1) was prepared by mixing equal quantities of each DNA-barcoded antibody and concentrating the panel on an Amicon Ultra 0.5 ml 30 kDa MWCO centrifugal filter (Millipore).

Live intact cells from control and double-mutant submandibular glands of ~P70 mice were prepared as described above. Cells from each animal were processed separately and immediately prepared for simultaneous transcriptome and epitope profiling as outlined in the online CITE-seq protocol (https://cite-seq.com/) using the panel of 63 barcoded antibodies. We further supplemented the cells with a low amount (3%) of human HEK cells (cultured and prepared as described before^18^) used as a spike-in control. The cells were then processed for Drop-seq and cDNA libraries prepared as described earlier. Antibody-derived tag (ADT) libraries were amplified for 12 cycles together with TruSeq Small RNA primers for indexing and sequenced together with cDNA libraries on Illumina NextSeq500 sequencers using the same settings as those used previously for single-cell transcriptome libraries.

### Immunostainings

Immunofluorescence analyses were performed on formalin-fixed paraffin-embedded tissue sections as described^76^. Antigen retrieval was accomplished by Tris-EDTA (10 mM Tris, 1 mM EDTA, 0.05% Tween-20, pH 9.0) at 99–100 °C for 20 min. Following retrieval, sections were stained with one or several of the following primary antibodies for immunodetection: mouse-anti-β-catenin (BD Transduction Laboratories, 610153), rabbit-anti-EGF (Abcam, ab9695), goat-anti-Smgc (Sigma, SAB2501988), rabbit-anti-Kal1 (aka Wfdc18; Abcam, Ab115270), rabbit-anti-Axin2 (Cell signalling, 2151), rabbit-anti-Wif1 (Abcam, ab186845), rabbit-anti-SMA (aka Acta2; Abcam, ab5694), goat-anti-Prol1 (Abcam, Ab119999), mouse-anti-PGRP (aka Pglyrp1; ThermoFisher, MA1-41044), rabbit-anti-Hepacam2 (Abcam, ab189943), goat-anti-Muc19 (Abcam, ab121014), mouse-anti-AQP5 (Santa Cruz Biotechnology, sc-514022), rabbit-anti-Clusterin (Abcam, ab92548), guinea pig-anti-CK8 (aka K8 or Krt8; Progen, GP-K8), rabbit-anti-CK14 antibodies (aka K14 or Krt14; ThermoFisher, MA5-11599), rabbit-anti-Klk1 (Boster, PA1709), mouse-anti-Ptn (Santa Cruz Biotechnology, SC74443), goat-anti-Sparc (R&D Systems; AF942). Secondary antibodies were conjugated with Cy2, Cy3, or Cy5 fluorochromes (Jackson ImmunoResearch Laboratories). Images were captured using an Axio imager Z1m and AxioCam MRm (Carl Zeiss) and a Leica TCS SP8.

### Processing and analysis of single-cell RNA-seq data

Drop-seq data were processed using Drop-seq tools v1.12^19^, based on the Gencode vM7 reference augmented by two pseudo-chromosomes containing the *Cre* and *EYFP* sequences, respectively. Selecting valid barcodes with the ‘knee’ method, resulting DGEs for each sample were combined and analyzed in R (version 3.4.4) using Seurat^21^ (version 2.3.4). We additionally filtered for at least 100 genes and less than 15% mitochondrial content. Clustering and t-SNE were performed based on a PCA with significant components chosen using JackStraw. For clustering, we used a resolution of 0.7 for the control samples and of 1.0 for the combination of all samples, and merged several acinar subclusters which were not distinguishable from their marker genes. We controlled replicate-to-replicate consistency (see below) across samples, and pooled and analyzed singe-cell datasets without any sample alignment method. For improved visualization, we additionally filtered out all cells located more than 3 standard deviations away from their cluster center and those which had a different identity than the majority of their 10 nearest neighbors in the t-SNE.

Bulk RNA-seq data were mapped to the same augmented Gencode vM7 reference using STAR^81^ (version 2.6.0c), quantified using featureCounts (version 1.6.0), and converted to TPM. For the comparison between bulk and single-cell RNA-seq, we used AverageExpression followed by a TPM transformation, and selected all genes found in all samples with TPM > 1.e−4.

For the CITE-seq samples, we used the same processing pipeline on the RNA fraction as for the other Drop-seq samples and filtered for at least 100 but less than 2500 genes and less than 15% mitochondrial content. We regressed out number of UMIs as well as the percentages of mitochondrial and ribosomal genes before performing clustering and t-SNE based on a PCA using 30 significant components. We also performed an additional analysis where we mapped against a combined hg19 and mm10 reference. From the second pipeline run we used reads mapping to the human genome to identify cells of human origin or doublets, and removed these cells from the standard pipeline output. We used CITEseq-count (v1.2) to quantify ADT counts for the respective cells found in both pipeline runs. Specifically binding antibodies were identified by inspecting ADT count distributions for human and mouse cells.

Batch effects and sample-to-sample variability were quantified using an entropy-based approach inspired by the one in Azizi et al.^20^. Specifically, we aimed to measure how well the local distribution of cells among samples mirrors the global one, taking into account that the latter might not be perfectly uniform. Therefore we used the relative entropy (Kullback-Leibler divergence) $D_{j}= \sum _{i\epsilon I}q_{i} \,log(q_{i}/q_{i}^{0})$, where *q*_*i*_ is the proportion of cells from group *i* in the local neighborhood *N(j)* of cell *j*, and $q_i^0$ is the proportion of cells of group *i* in the entire data set. The set *I* of sample groups was defined by the biological factors sex, stage, and genotype, by their combination, by technical factors such as cell dissociation protocol, or simply by replicate, and the local neighborhood was defined by taking the 30 nearest neighbors in the kNN-Graph (calculated in PCA space). Controls were obtained by randomly shuffling the group assignment between cells.

The relative density of two sample groups on the t-SNE was plotted using the log2 ratio of two separate 2D kernel density estimators interpolated on the t-SNE coordinates of each cell. Differences in cluster proportions were analyzed using mixed-effects linear models (lme4 package;^82^ version 1.1) using a binomial model with sex, stage and genotype (if applicable) as fixed effects and sample identity as random effect.

Subclustering of cancer stem and basal cells was performed on these populations separately, removing one sample (B9T90R4F) with high content of ribosomal genes and the few cells from control samples from the cancer stem cell clusters. Differential genes between subclusters were detected by pooling counts over all cells from one sample and subcluster, using DESeq2^83^ (version 1.18) on the pooled counts with the number of cells per group as covariate, and then filtering for genes expressed in at least 5% of cells. Pathway analysis was done with GAGE^84^ (version 2.28) on the log2 fold changes estimated by DESeq2. We used HALLMARK, KEGG and REACTOME gene sets from the Molecular Signature Database (MsigDB: http://www.broad.mit.edu/gsea), kept pathways with a *q*-value <0.1 and absolute average log2 fold changes >0.5, and ignored those with more than 10% ribosomal genes. For the diffusion map, we combined the tumor-specific basal and CSC subclusters with the luminal Clu+ cluster and used destiny^85^ (version 2.6.2) on the highly variable genes to create the diffusion map embedding and diffusion pseudotime. We also ran destiny on each sample with more than 10 cells in the relevant subpopulations separately and compared sample-specific to global pseudotime estimates. Differential genes along the trajectory were identified on pooled counts using DESeq2 with a likelihood ratio test on all 4 clusters simultaneously, and gene expression values plotted along the trajectory after imputation using SAVER^86^ (version 1.1.1). Gene set scores were computed supplying imputed expression values to Seurat’s AddModuleScore function. An additional “stemness” gene set (*Pou5f1*, *Nanog*, *Sox2*, *Prom1*, *Bmi1*, *Lgr5*, *Msi1*, *Tdgf1*, *Bmp4*, *Cspg4*, *Cxcr4*, *Alcam*, *Slc2a13*, *Aldh1a*) was curated from the literature^64^.

### Reporting summary

Further information on research design is available in the Nature Research Reporting Summary linked to this article.

## Supplementary information

Supplementary Information

Description of Additional Supplementary Files

Supplementary Data 1

Reporting Summary

## Data Availability

The sequencing data generated is available in GEO under the accession GSE124425. Metadata for each sequenced cell, from which figures based on the single-cell RNA sequencing data can be reproduced, are provided as a source data file.

## Source Data

Source Data

## Code availability

Custom R scripts used to analyze data and generate figures are available upon request.
