# Supplementary material for: Tracing tumorigenesis in a solid tumor model at single-cell resolution
Source: Nat Commun. 2020 Feb 20;11:991. doi: 10.1038/s41467-020-14777-0 (PMC7033116; doi:10.1038/s41467-020-14777-0)
Supplement: Supplementary file 1 — Supplementary Information [file 41467_2020_14777_MOESM1_ESM.pdf]

Supplementary Information

Tracing tumorigenesis in a solid tumor model at single-cell resolution

Praktiknjo et al.

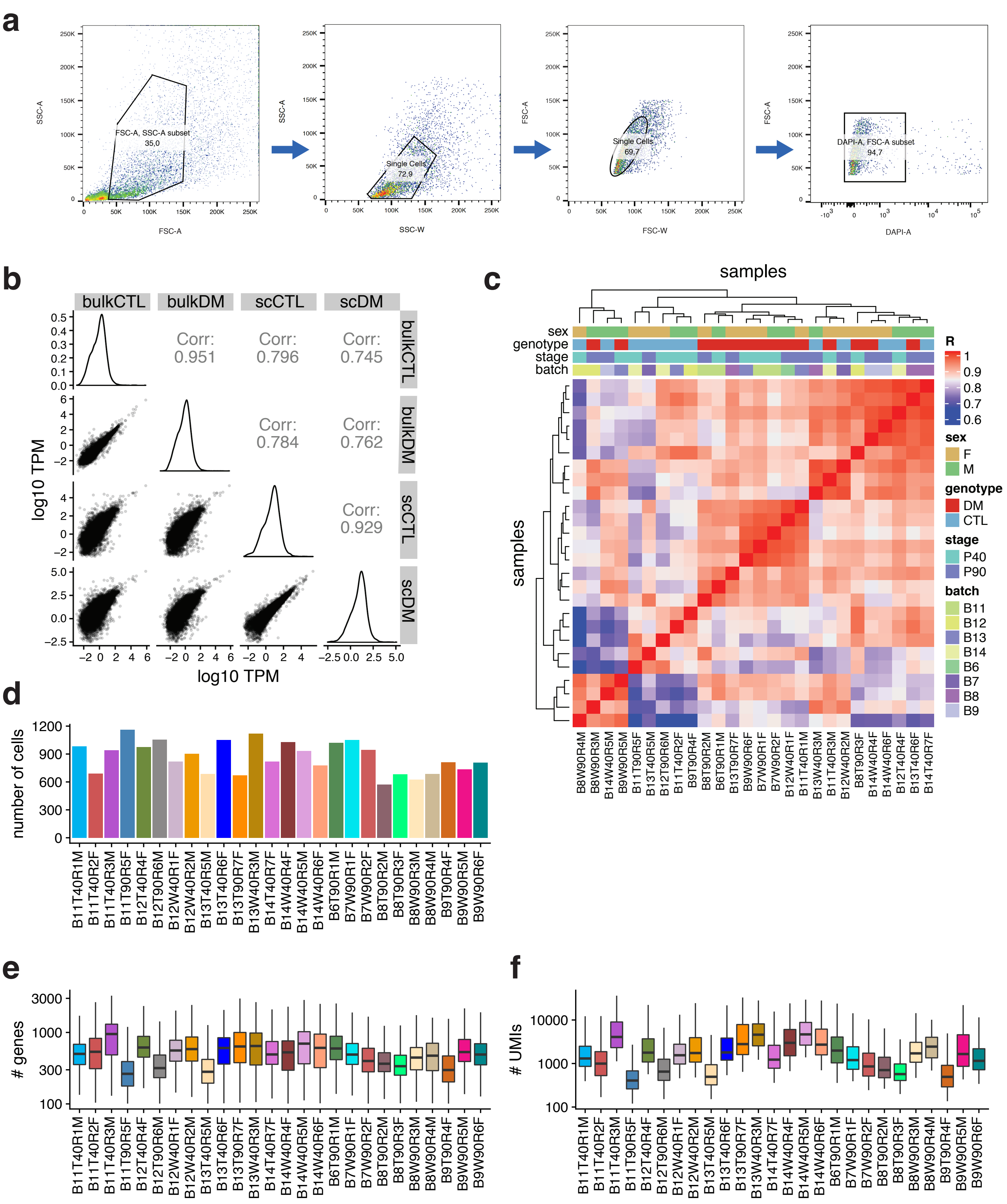

Supplementary Fig. 1

Supplementary Fig 1. Single-cell sample preparation and run statistics. a Flow cytometric approach to obtain live, single cells, from dissociated submandibular salivary glands. Cells were first sorted by size to remove cellular debris and doublets, and the subset of DAPI-negative cells further selected to exclude dead cells. **b** Pairwise gene expression correlations between samples pooled from single-cell control (scCTL) or single-cell double-mutant (scDM) datasets and bulk RNA-seq data from freshly dissected whole control (bulkCTL) or double-mutant (bulkDM) submandibular glands. *Lower left part*: correlation scatter plots where axes correspond to either single-cell expression counts (UMIs) converted to log10-transformed average transcripts per million (ATPM) or RNA-seq bulk expression levels in transcripts per million (TPM). *Upper right part*: Pearson correlation coefficients for the respective comparison. *Diagonal*: expression density distribution plots of samples. **c** Correlation heatmap of normalized expression values (as described above) for the 26 individual single-cell libraries. Pearson coefficients were generally high as indicated by a color scale ranging from blue (R=0.6) to red (R=1). Samples did not cluster in an obvious way according to the sex (F=female vs. M=male), genotype (control vs. double-mutant), stage (P40 vs. P90) of the animal or the experimental batch in which they were processed. **d** Mean number of cells obtained per sample after single-cell experimental and computational processing. **e** Box plots showing the distribution of genes per cell for each sample. **f** Box plots showing the distribution of the number of UMIs per cell for each sample. **e, f** Boxes span the 25th to the 75th percentile, whiskers 1.5 times the interquartile range. Cell number per sample as indicated in d.

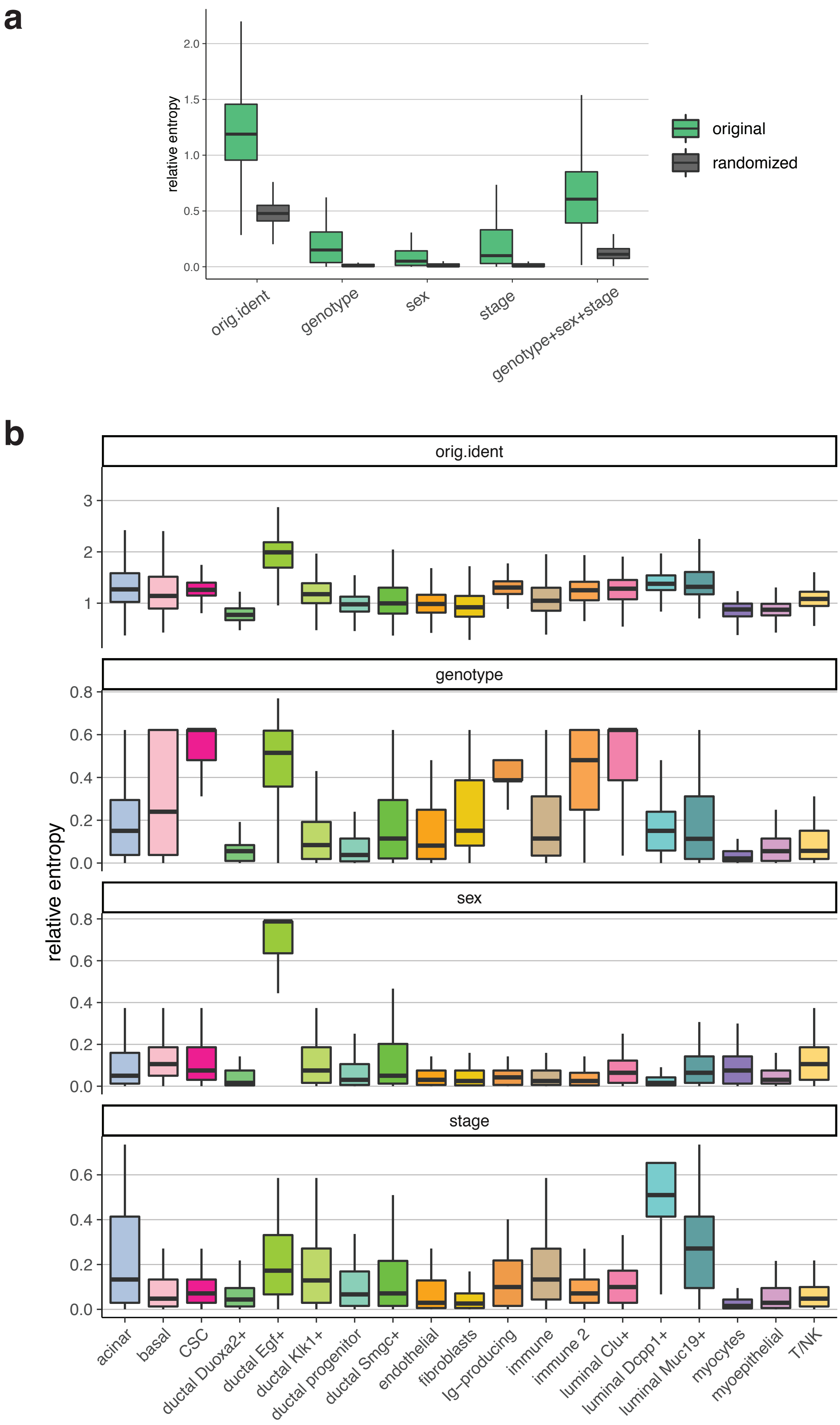

Supplementary Fig. 2

Supplementary Fig 2. Entropy-based quantification of batch effects. We used relative entropy (Kullback-Leibler divergence) to quantify how well a cell's local neighborhood reflects the global distribution of cells across samples or other biological groups, such as genotype, stage, sex or the combination of all three. 22525 cells in 26 samples used in total. **a** Distribution of relative entropy values for cells grouped by sample (orig.ident), genotype, sex, stage or the combined effect of genotype+sex+stage, compared to randomizing these labels across cells. **b** Relative entropy per cell type is highest in cell types that exhibit the strongest differences related to a specific biological factor (e.g., CSCs for genotype, ductal Egf+ for sex or luminal Dcpp1+ for stage). **a, b** Boxes span the 25th to the 75th percentile, whiskers 1.5 times the interquartile range.

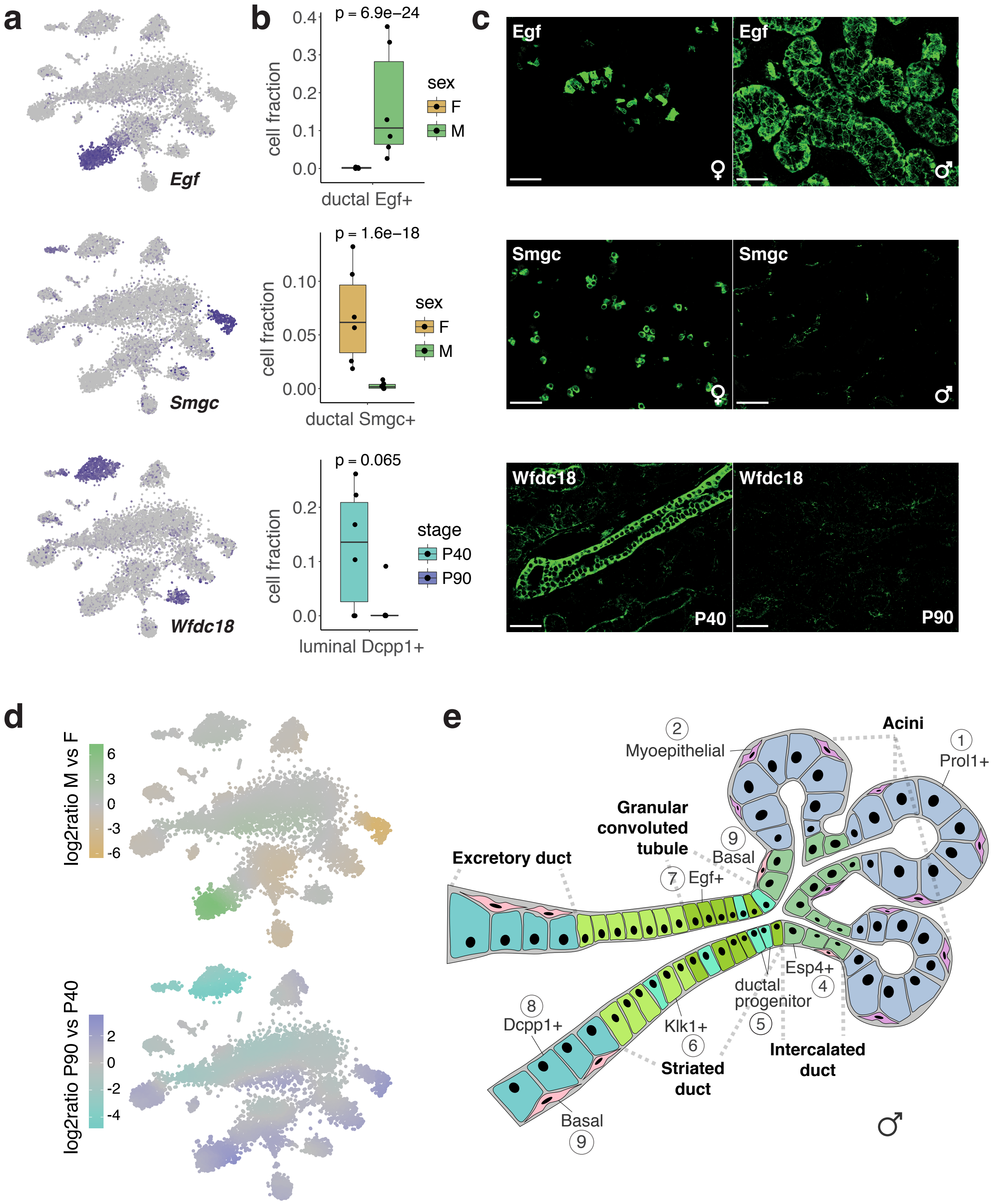

Supplementary Fig. 3

Supplementary Fig 3. Validation of sex- and stage-specific cell clusters. a Expression of *Egf* (top), *Smgc* (middle) and *Wfdc18* (bottom) in the tSNE representation as indicated by a color scale ranging from grey (no expression) to dark blue (high expression). **b** Fraction of cells from male and female (top and middle) or P40 and P90 (bottom) animals contributing to the ductal Egf+, ductal Smgc+, and luminal Dcpp1+ clusters. Boxes span the 25th to the 75th percentile, whiskers 1.5 times the interquartile range. P-values from mixed-effects binomial model using 10398 cells in 12 samples. **c** Immunofluorescence analysis of submandibular gland tissue sections from female and male (top and middle) and P40 and P90 (bottom) animals for Egf, Smgc and Wfdc18 proteins. Representative images of at least two independent experiments. Scale bars: 50 µm. **d** tSNE plots showing either sex- (top) or stage-specific (bottom) relative local densities of cells as determined by the log2-transformed ratio of male to female (green to dark yellow) or P90 to P40 (blue to turquoise) contributions in neighbouring cells, respectively. **e** Anatomical sketch of the male submandibular gland based on single-cell transcriptome data, available literature (see text for references) and validations in tissue sections by immunofluorescence.

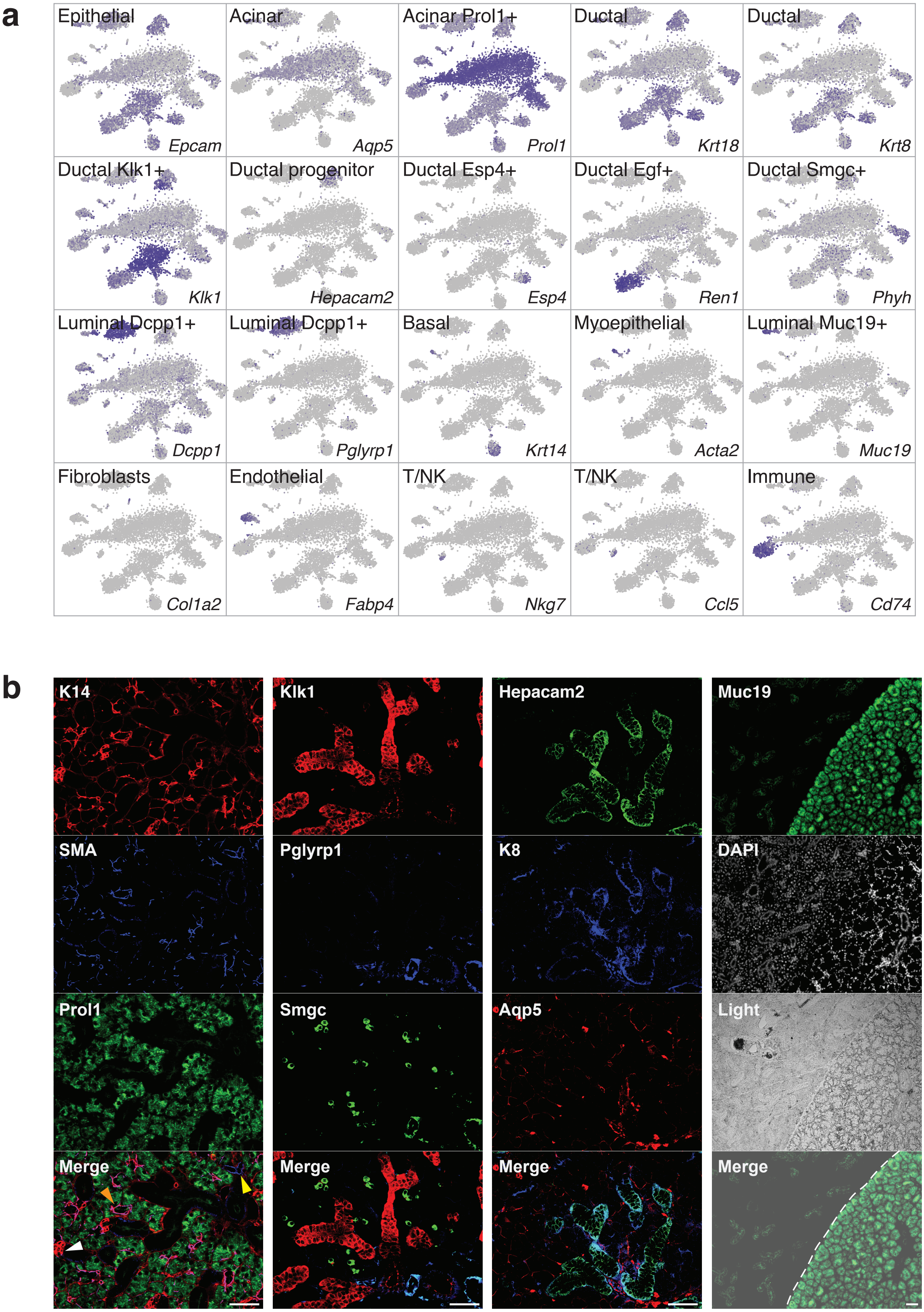

Supplementary Fig. 4

Supplementary Fig 4. Cell type-specific marker expression in control salivary glands. a Projection of several differentially expressed genes in clusters onto the tSNE showing some of the markers which were used for the cell type annotation. Expression levels are indicated by a color scale ranging from grey (no expression) to dark blue (high expression). **b** Validation of cell type-specific epithelial markers by immunofluorescence analysis in salivary gland tissue sections from control animals. From left to right, (co)localizations of: K14 (basal), SMA (myoepithelial), Prol1 (acinar) / Klk1 (striated), Pglyrp1 (excretory), Smgc (intercalated ductal in females) / Hepacam2 (ductal progenitor), K8 (ductal), Aqp5 (acinar) positive cells in submandibular glands; and Muc19-positive cells in the sublingual gland. White, yellow and orange arrowheads mark K14-high, SMA-high and combined K14/SMA-high cells, respectively. Dashed line the boundary between submandibular (left) and sublingual (right) tissue. Except for Muc19, representative images of at least two independent experiments. Scale bars: 50 μm.

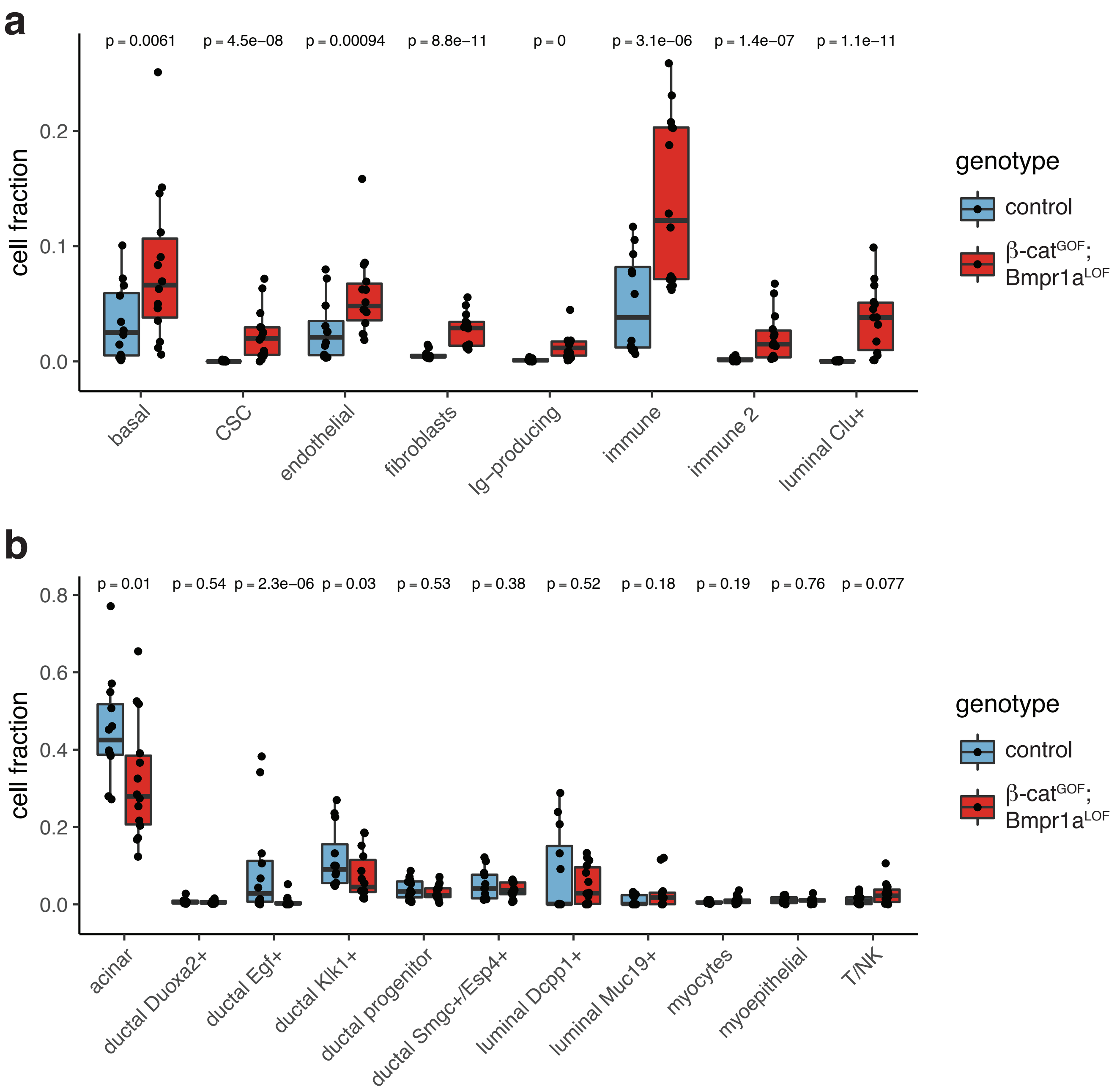

Supplementary Fig. 5

Supplementary Fig. 5. Proportions of enriched cell populations in double-mutant tissues. CSCs, basal, luminal Clu+, immune, immune2, Ig-producing and stromal (fibroblasts and endothelial) cell clusters were significantly more abundant in tumor-bearing tissues (**a**), while other prominent epithelial cell populations were accordingly more abundant in the control (**b**). P-values from mixed-effects model using 22525 cells in 26 samples. Boxes span the 25th to the 75th percentile, whiskers 1.5 times the inter-quartile range.

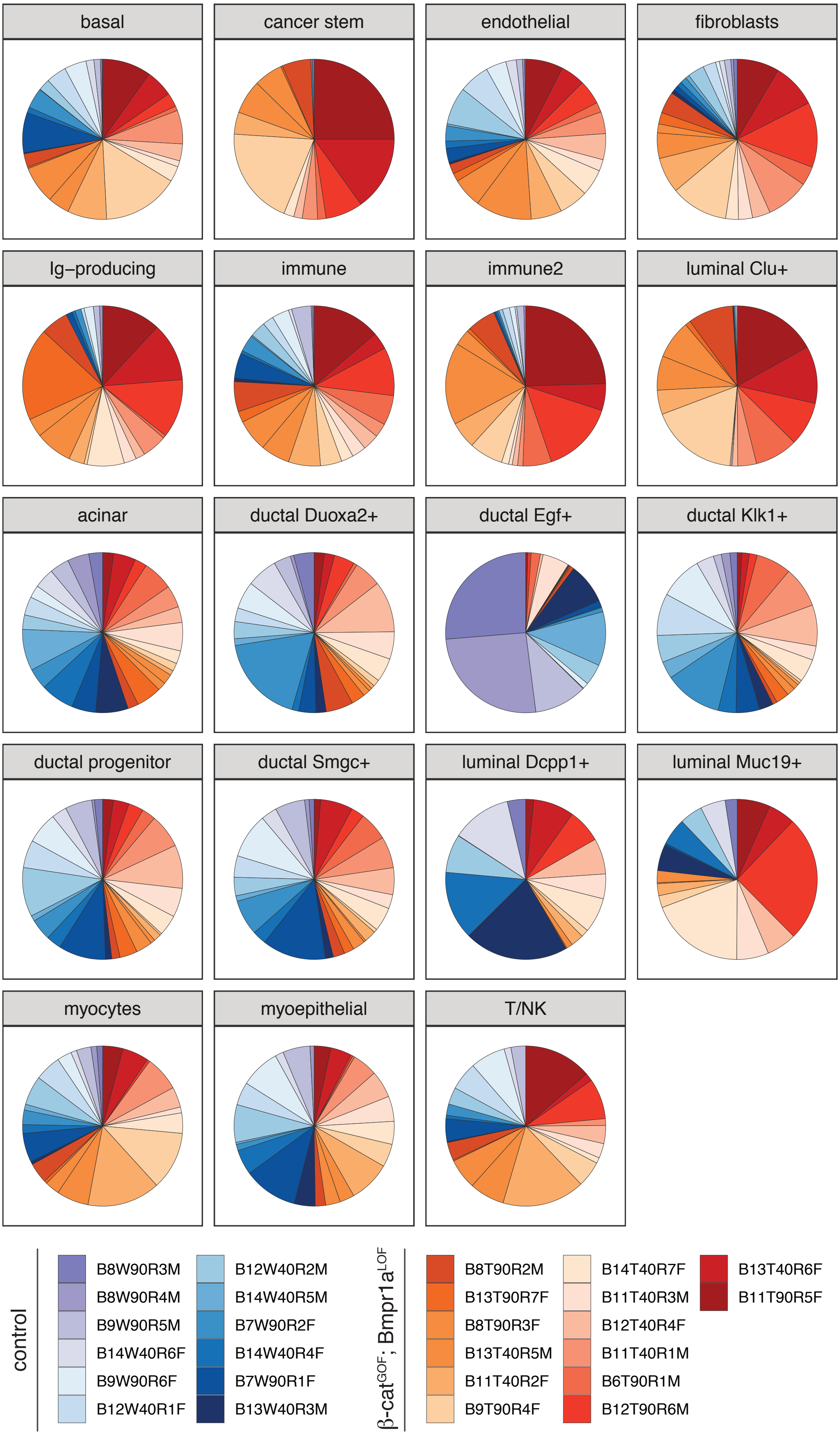

Supplementary Fig. 6

Supplementary Fig. 6. Relative contributions of samples to cell clusters. Control samples are shown in shades of purple and blue; double-mutant tumor-bearing samples in shades of red and orange.

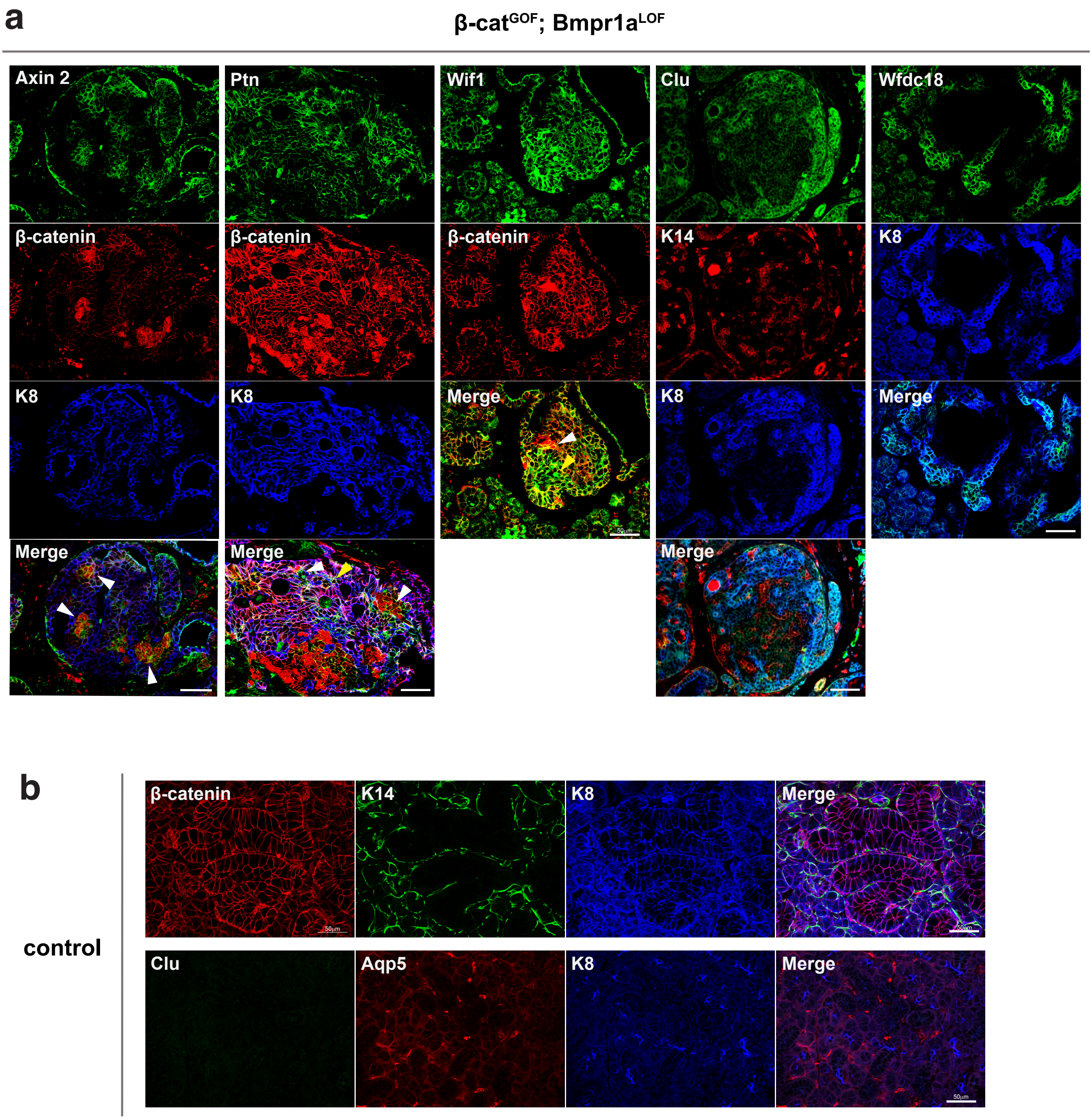

Supplementary Fig. 7

Supplementary Fig. 7. Immunofluorescence analysis of tumor-specific markers in double-mutant and control tissues. a Validation of tumor-specific cells (cancer stem, tumor-specfic basal and luminal Clu+ cells) by immunofluorescence in tumor regions of double-mutant salivary gland tissue sections using markers identified from our single-cell data (see Fig. 2c). White arrowheads indicate co-staining of Axin2, Ptn and Wif1 with nuclear β-catenin. Yellow arrowheads indicate co-staining of Ptn with K8 or point out Wif1-positive cells. Clu and Wfdc18 stainings strongly correlated with K8-positive cells in tumor lesions. **b** Co-stainings of β-catenin/K14/K8 and Clu/Aqp5/K8 in tissue sections from control salivary glands show absence of nuclear β-catenin and Clu-positive cells. **a, b** Representative images of at least two independent experiments. Scale bars: 50 µm.

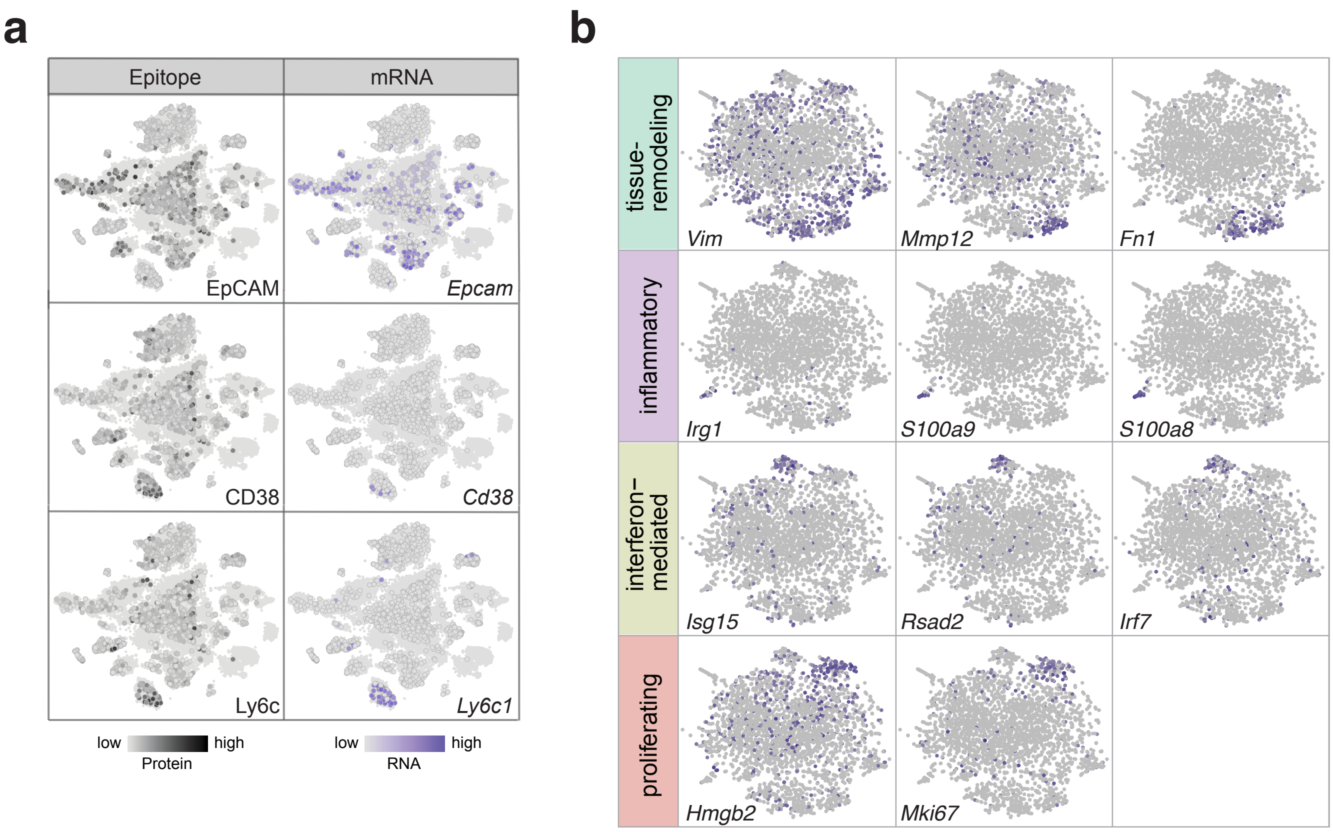

Supplementary Fig. 8

Supplementary Fig. 8: Cell surface markers and expressed genes in the tumor microenvironment. **a** Epitope and mRNA signals in cells from CITE-seq experiments for epithelial (Epcam) and stromal-specific markers (Cd38, Ly6c) in the tSNE of the combined clustering as shown in Fig. 3b. **b** Gene expression of several markers in the subclustered tSNE representation of immune cells as shown in Fig. 3d.

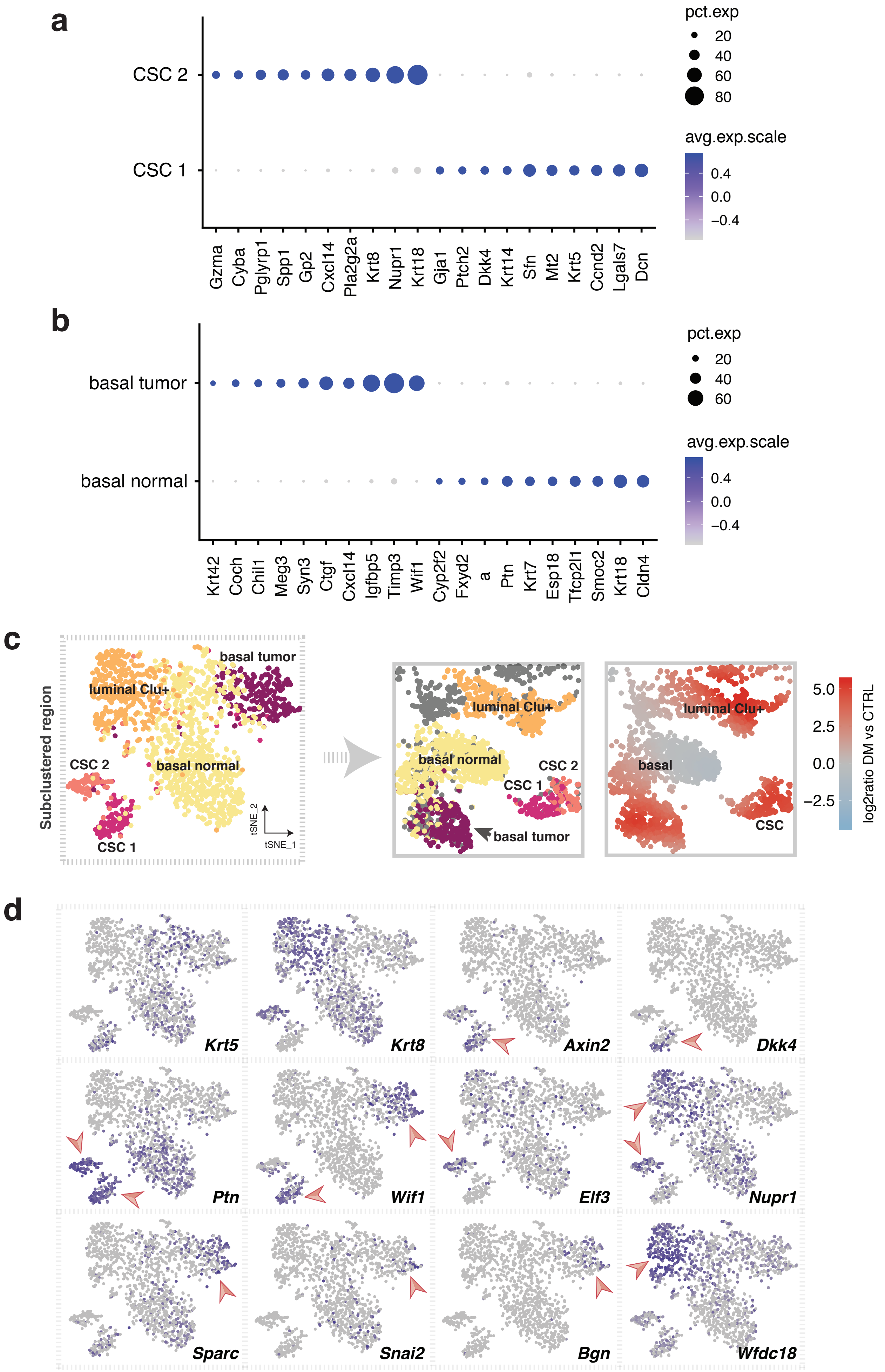

Supplementary Fig. 9

Supplementary Fig. 9: **Characterization of CSC-like and basal subpopulations**. **a, b** Top marker genes for CSC (**a**) and basal (**b**) subpopulations, respectively. **c** Projection of the subclustered tumor-specific region back onto a subregion of the tSNE shown in Fig. 4a. **d** Expression of several genes in the subclustered tumor-specific tSNE representation as indicated by a color scale ranging from grey (no expression) to dark blue (high expression).

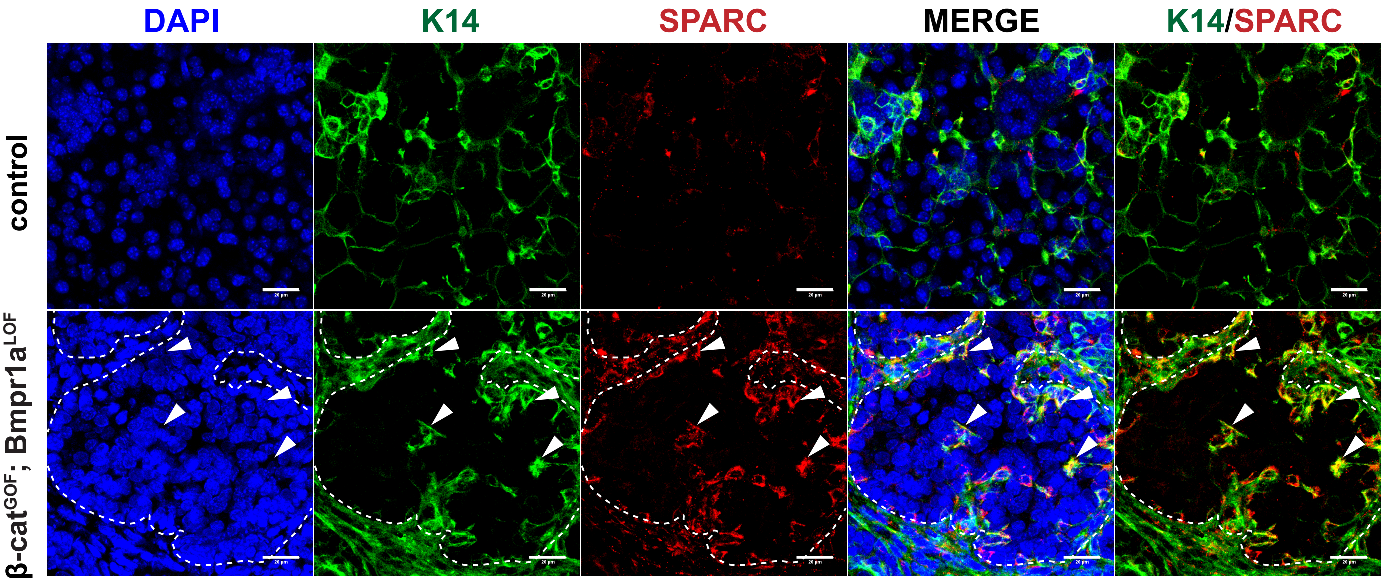

Supplementary Fig. 10

Suppementary. Fig. 10: EMT occurs in basal cells at early stages of tumorigenesis. Immunofluorescence of control (top) and double-mutant (bottom) salivary glands at P40 with antibodies against SPARC, an emerging EMT marker that is associated with tumor progression and increased invasiveness in several cancers (see supplemental references[1-5]). SPARC was particularly prevalent in the tumor region of double-mutant tissues. Compared to control tissue, SPARC was upregulated in K14-positive cells within the tumor as well as in the tumor-associated stroma. Arrows indicate co-staining of K14 and SPARC. Dashed lines indicate the tumor region in the double-mutant tissue section. Representative images of two independent experiments. Scale bar: 20 µm.

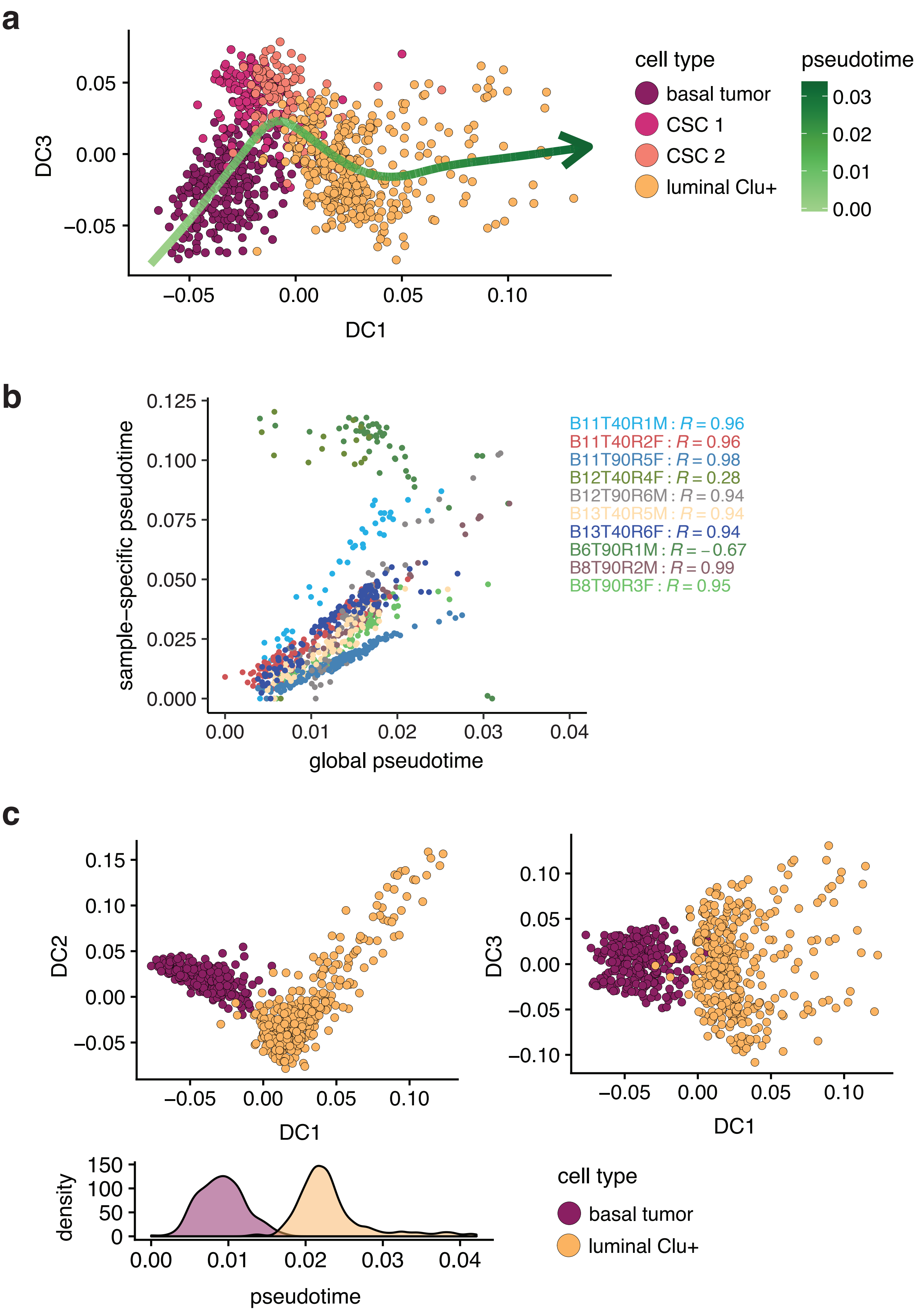

Supplementary Fig. 11

Supplementary Fig. 11: **Diffusion map control analyses. a** Diffusion map of tumor-specific epithelial cell populations shown in the first and third components together with inferred trajectory obtained by smoothing diffusion coordinates over pseudotime. **b** Diffusion analysis for individual double-mutant tumor samples with >10 cells in the relevant clusters showing sample-specific pseudotime plotted against global pseudotime. With the exception of B6T90R1M (dominated by luminal Clu+ cells) and B12T40R4F (only 15 cells) correlations (Pearson's R) were generally high. **c** Diffusion map shown in the first and second (left) and first and third (right) components of tumor-specific epithelial cell populations after removing CSCs from the analysis. Respective density plot of basal tumor and luminal Clu+ cells along pseudotime is shown (bottom).

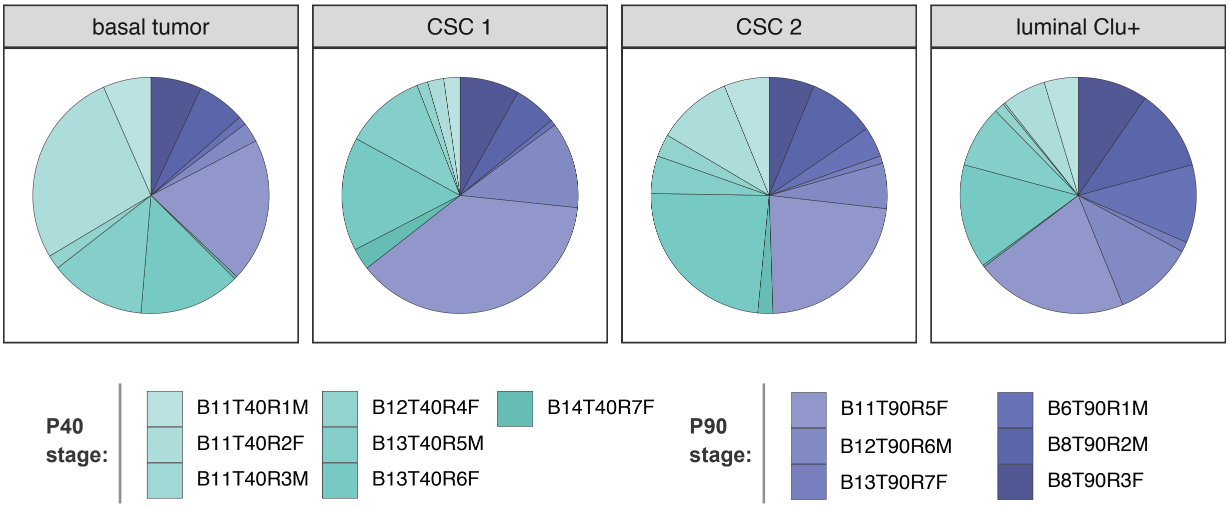

Supplementary Fig. 12

Supplementary Fig. 12: Relative contributions of double-mutant samples to tumor-specific epithelial cell types. Samples from the P40 and P90 stage are shown in shades of turquoise and dark blue, respectively.

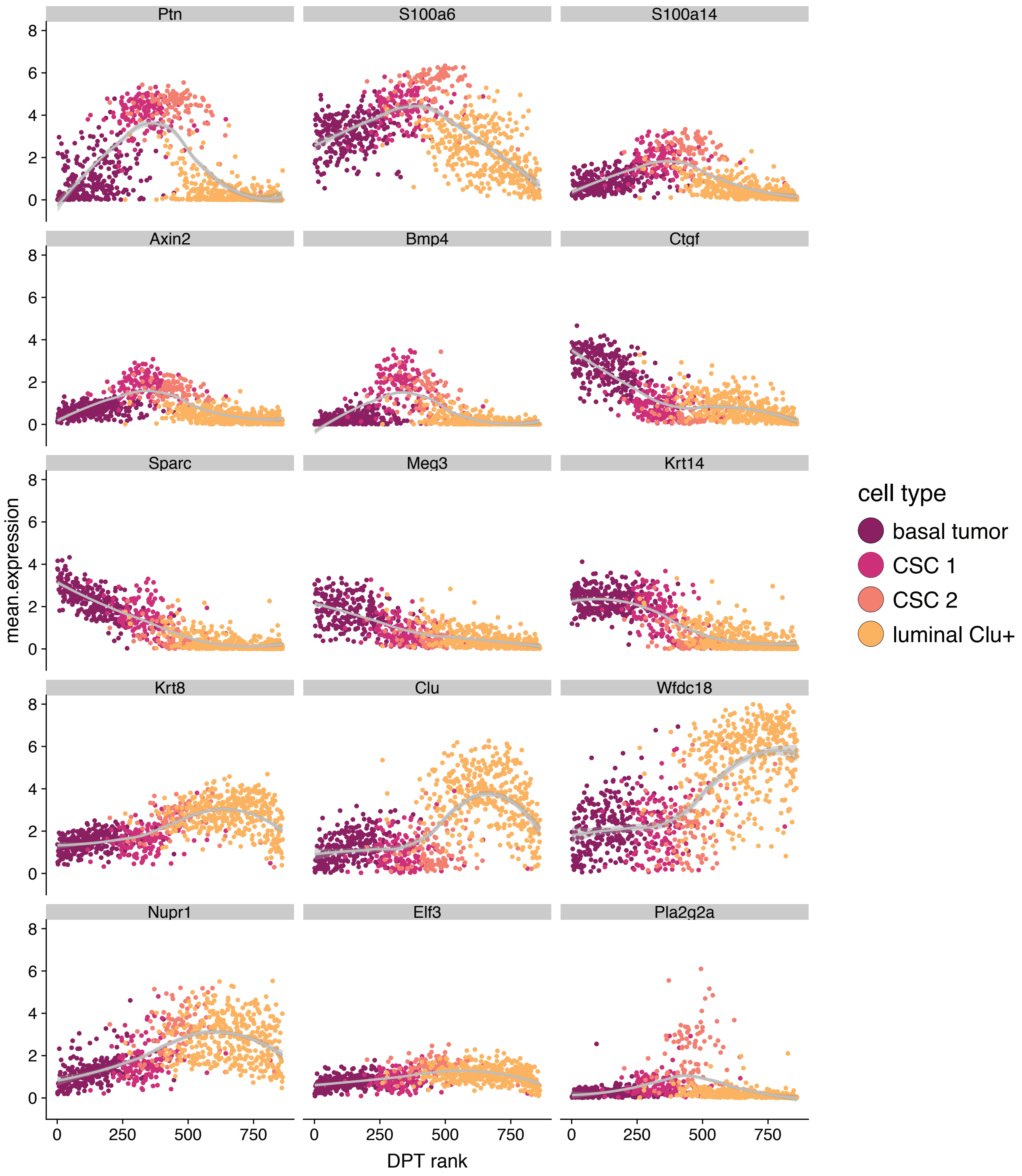

Supplementary Fig. 13

Supplementary Fig. 13: Gene expression for selected differential genes as function of pseudotime rank. SAVER-imputed gene expression with grey lines from LOESS smoothing (shading indicates 95% confidence interval).

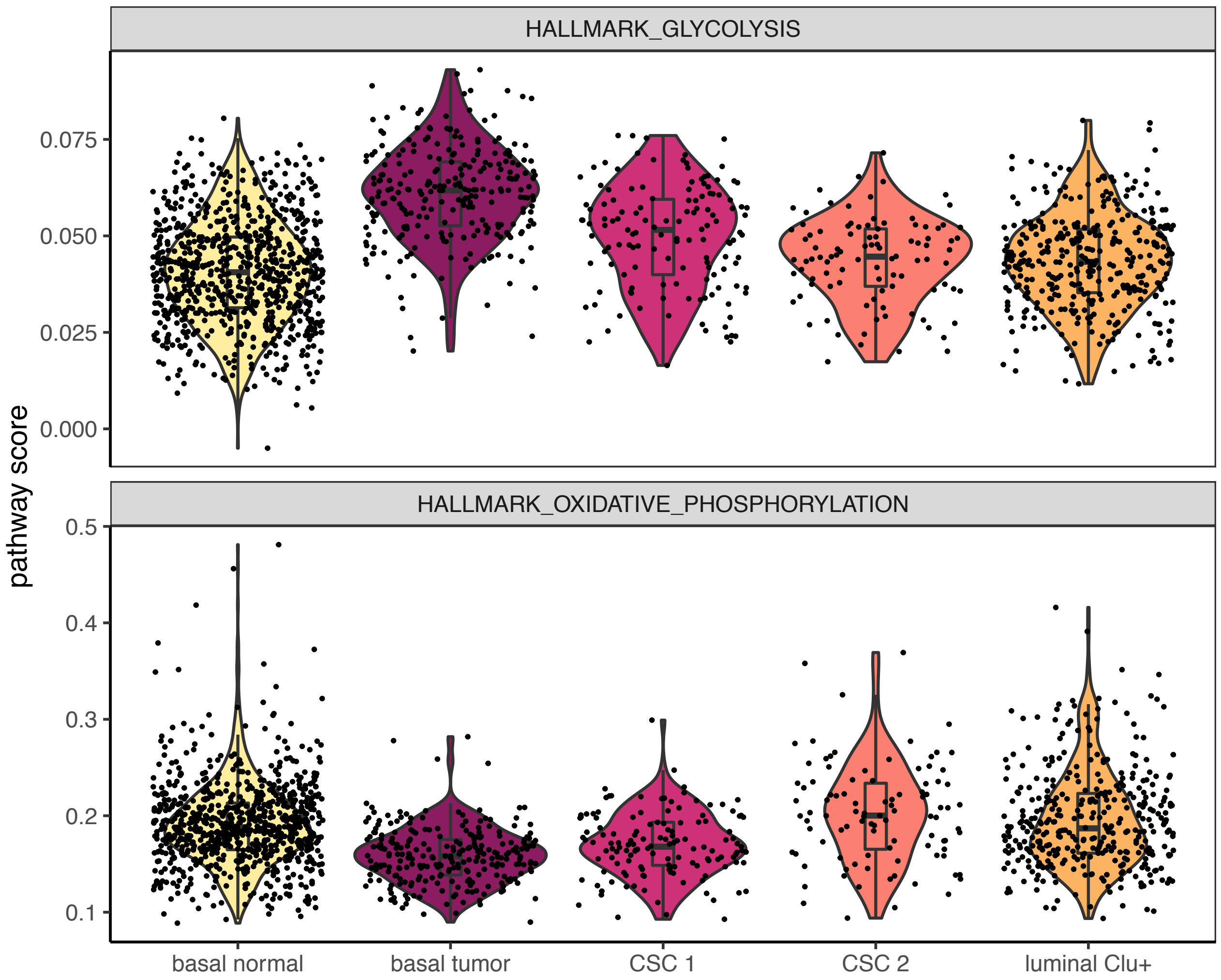

Supplementary Fig. 14

Supplementary Fig. 14. Metabolic pathway scores in tumor-specific epithelial cells. Violin plots with boxplots showing the distribution of HALLMARK_GLYCOLYSIS and HALLMARK_OXIDATIVE_PHOSPHORYLATION gene set scores from SAVER-imputed expression data in tumor-specific epithelial cells as defined in Fig. 4 compared to the 'basal normal' subpopulation (shown on the left in yellow).

Antibody	Clone	CITE-seq barcode	Antibody	Clone	CITE-seq barcode	Antibody	Clone	CITE-seq barcode
CD62L	MEL-14	AATCGCTCCGGA	CD54	YN1/1.7.4	GGACATTACCAC	control_mIgG2b	27-35	GATCGTAATACC
A2B5	105	AGCGAAGACGAT	CD74	In1/CD74	TATACGGACGTG	Nectin2*		AACCATGGTCGC
control_AH_IgG	HTK888	CATGATTGGCTC	CD69	H1.2F3	ACGGCTAATCAC	NK1.1	PK136	AGCAAGCCTCAT
CCR9_CD199	L053E8	ACCGATCTCAGC	CD71	RI7217	TAGGCTGCTTAA	Notch1	HMN1-12	GCTCAGATTAGT
CD103	Clone 2E7	CCGCGTTACACA	CD8	5H10	CCGATCGTATGC	Notch2	HMN2-35	CATACGCGAAGG
CD104	346-11A	CTTAACTCATGG	CD95	SA367H8	ATCTATGCCTCC	Notch3	HMN3-133	TGCTGAGGTCTT
CD11b	M1/70	TCAATTGCGTGC	CD97	18d3	AACGTAACTGAG	Notch4	MHN4-2	GCGTCCGAGAAT
CD11c	N418	CGTAAGAACCGT	cKit	2B8	CCTCGGATACTA	PDCA1_CD137	17B5	TTCGTACAGTTC
CD127	A7R34	CGTACAAGCCAC	CLEC12A_CD371	5D3/CLEC12A	GAACTTCTGGCG	SiglecF	S17007L	CGAAGAGGCCTT
CD133	315-2C11	CCAATACGAGCA	CLEC9A_DNGR1_CD370	7H11	TGAGCCTCACTT	SiglecH	551	CGTGATTGAAGG
CD152	9H10	CTGACGACTCAG	CSF1R_CD115	AFS98	TTGATCGACCGT	SIRPa_CD172a	P84	GAGTAGCACATA
CD16_32	93	CAGTTGCTCTGA	CX3CR1	SA011F11	GTTGTTGGTCCG	TCRg_d	GL3	GCGACAATGACG
CD169	3D6.112	CTAGCTGACGCA	Delta_Like_4	HMD4-1	AGGCTAAGGCAA	TNFRH3_TNFRSF26*		TCTCTCAAGTCC
CD19	1D3	CATGTCTACATC	EpCAM	G8.8	GAGGACGATCAT	Trop2*		GAACTCATAGGC
CD205	NLDC-145	TCTGGAGGACAA	ESCAM*		ATAGGTCATGCG	XCR1	ZET	GTCCAACAGCCA
CD209a	MMD3	CAATAGCAGCTC	IAIE_MHC	M5/114.15.2	TGGCTGGCTAGA	Jagged2	HMJ2-1	GTGGATCATGTT
CD24	M1/69	TAGTGCTAGGCG	IL7Ra_CD127	A7R34	CGGAGTAGTAAT	CD29	HM_1-1	TTCACTGGCTAA
CD25	3C7	AACTGCTCCACA	Lamp1	1D4B	GAACTCCACCTC			
CD317	REA818	GTCTGTAGGCAT	Ly6A/E	D7	ATGCCAGCAGAG			
CD34	HM34	GTTGAAGACTGG	Ly6C	HK1.4	AAGAGCTCGCAG			
CD38	90	GGCCGAGTCTAA	Ly6G	1A8	TCGATAACCGCT			
CD4	RM4-5	TGACGTAACACT	control_mIgG1	MG1-45	TGTCCGGCAATA			
CD45R	RA3-6B2	ACGAGGAGATGG	control_mIgG2a	MG2a-53	GAGGCGATTGAT			

Supplementary Table 1: List of oligonucleotide-coupled antibodies used for CITE-seq experiments. All uncoupled antibodies were obtained from BioLegends except for those indicated by an asterisk which were obtained from R&D Systems.

Supplementary References

1 Framson, P. E. & Sage, E. H. SPARC and tumor growth: where the seed meets the soil? *Journal of cellular biochemistry* **92**, 679-690, (2004).

2 Feng, J. & Tang, L. SPARC in Tumor Pathophysiology and as a Potential Therapeutic Target. *Current pharmaceutical design* **20**, 6182-6190, (2014).

3 Grant, J. L. *et al.* A novel molecular pathway for Snail-dependent, SPARC-mediated invasion in non-small cell lung cancer pathogenesis. *Cancer prevention research (Philadelphia, Pa.)* **7**, 150-160, (2014).

4 Girotti, M. R. *et al.* SPARC promotes cathepsin B-mediated melanoma invasiveness through a collagen I/alpha2beta1 integrin axis. *The Journal of investigative dermatology* **131**, 2438-2447, (2011).

5 Fenouille, N. *et al.* The epithelial-mesenchymal transition (EMT) regulatory factor SLUG (SNAI2) is a downstream target of SPARC and AKT in promoting melanoma cell invasion. *PloS one* **7**, e40378, (2012).
