# Supplementary material for: Tracing tumorigenesis in a solid tumor model at single-cell resolution
Source: Nat Commun. 2020 Feb 20;11:991. doi: 10.1038/s41467-020-14777-0 (PMC7033116; doi:10.1038/s41467-020-14777-0)
Supplement: Supplementary file 2 — Description of Additional Supplementary Files [file 41467_2020_14777_MOESM2_ESM.pdf]

Description of Additional Supplementary Files

File Name: Supplementary Data 1

Description: Differential genes along the trajectory. List of differentially expressed genes at the 4 boundaries between basal normal, basal tumor, CSC 1, CSC 2 and luminal Clu+ at 5% FDR.
