# Supplementary material for: Tracing tumorigenesis in a solid tumor model at single-cell resolution
Source: Nat Commun. 2020 Feb 20;11:991. doi: 10.1038/s41467-020-14777-0 (PMC7033116; doi:10.1038/s41467-020-14777-0)
Supplement: Supplementary file 4 — Reporting Summary [file 41467_2020_14777_MOESM4_ESM.pdf]

nature research

Corresponding author(s): Samantha Praktiknjo, Walter Birchmeier, Nikolaus Rajewsky

Last updated by author(s): Jan 22, 2020

Reporting Summary

Nature Research wishes to improve the reproducibility of the work that we publish. This form provides structure for consistency and transparency in reporting. For further information on Nature Research policies, see Authors & Referees and the Editorial Policy Checklist.

Statistics

For all statistical analyses, confirm that the following items are present in the figure legend, table legend, main text, or Methods section.

n/a	Confirmed	
☐	☒	The exact sample size (n) for each experimental group/condition, given as a discrete number and unit of measurement
☐	☒	A statement on whether measurements were taken from distinct samples or whether the same sample was measured repeatedly
☐	☒	The statistical test(s) used AND whether they are one- or two-sided<br>*Only common tests should be described solely by name; describe more complex techniques in the Methods section.*
☐	☒	A description of all covariates tested
☐	☒	A description of any assumptions or corrections, such as tests of normality and adjustment for multiple comparisons
☐	☒	A full description of the statistical parameters including central tendency (e.g. means) or other basic estimates (e.g. regression coefficient) AND variation (e.g. standard deviation) or associated estimates of uncertainty (e.g. confidence intervals)
☐	☒	For null hypothesis testing, the test statistic (e.g. F, t, r) with confidence intervals, effect sizes, degrees of freedom and P value noted<br>*Give P values as exact values whenever suitable.*
☒	☐	For Bayesian analysis, information on the choice of priors and Markov chain Monte Carlo settings
☒	☐	For hierarchical and complex designs, identification of the appropriate level for tests and full reporting of outcomes
☐	☒	Estimates of effect sizes (e.g. Cohen's d, Pearson's r), indicating how they were calculated

Our web collection on statistics for biologists contains articles on many of the points above.

Software and code

Policy information about availability of computer code

Data collection	bcl2fastq (v2.20.0)<br>Drop-seq tools (v1.12)
Data analysis	R (v3.4.4) with packages Seurat (v2.3.4), lme4 (v1.1), DESeq2 (v1.18), GAGE (v2.28), destiny (v2.6.2), SAVER (v1.1.1)<br>STAR (v2.6.0)<br>featureCounts (v1.6.0)<br>CITEseq-count (v1.2)<br>Analysis scripts and R markdowns will be provided upon request

For manuscripts utilizing custom algorithms or software that are central to the research but not yet described in published literature, software must be made available to editors/reviewers. We strongly encourage code deposition in a community repository (e.g. GitHub). See the Nature Research guidelines for submitting code & software for further information.

Data

Policy information about availability of data

All manuscripts must include a data availability statement. This statement should provide the following information, where applicable:

- Accession codes, unique identifiers, or web links for publicly available datasets
- A list of figures that have associated raw data
- A description of any restrictions on data availability

The sequencing data generated is available in GEO under the accession GSE124425 (http://www.ncbi.nlm.nih.gov/geo/query/acc.cgi?acc=GSE124425)

October 2018

Field-specific reporting

Please select the one below that is the best fit for your research. If you are not sure, read the appropriate sections before making your selection.

☒ Life sciences ☐ Behavioural & social sciences ☐ Ecological, evolutionary & environmental sciences

For a reference copy of the document with all sections, see nature.com/documents/nr-reporting-summary-flat.pdf

Life sciences study design

All studies must disclose on these points even when the disclosure is negative.

Sample size	We analyzed the transcriptomes of > 22,000 single cells. Selection of cell barcodes and filtering were performed as described in the methods using Drop-seq tools (v1.12) and Seurat (v2.3.4).
Data exclusions	No data was excluded.
Replication	Transcriptome data from > 22,000 single cells, obtained from at least 3 mice of each experimental group.
Randomization	Clustering analysis showed that data was not influenced by experimental batch.
Blinding	Although mouse genotypes were known, data collection and analyses were performed unbiasedly.

Reporting for specific materials, systems and methods

We require information from authors about some types of materials, experimental systems and methods used in many studies. Here, indicate whether each material, system or method listed is relevant to your study. If you are not sure if a list item applies to your research, read the appropriate section before selecting a response.

Materials & experimental systems

n/a	Involved in the study	
☐	☒	Antibodies
☒	☐	Eukaryotic cell lines
☒	☐	Palaeontology
☐	☒	Animals and other organisms
☒	☐	Human research participants
☒	☐	Clinical data

Methods

n/a	Involved in the study	
☒	☐	ChIP-seq
☐	☒	Flow cytometry
☒	☐	MRI-based neuroimaging

Antibodies

Antibodies used	Antibodies used in the study are listed in the Methods and Supplementary Information.
Validation	Antibody-specific validations are available as indicated on the manufacturers' webpage.

Animals and other organisms

Policy information about studies involving animals; ARRIVE guidelines recommended for reporting animal research

Laboratory animals	All mice used in this study had a C57BL/6 background. Control and double-mutant mice were obtained as described in the Methods. Female and male mice staged around P40, P70 and P90 were collected for experiments.
Wild animals	The study did not involve wild animals.
Field-collected samples	The study did not involve samples collected from the field.
Ethics oversight	Animal experiments were approved by LAGeSo Berlin and performed according to EU and national institutional regulations

Note that full information on the approval of the study protocol must also be provided in the manuscript.

Flow Cytometry

Plots

Confirm that:

☒ The axis labels state the marker and fluorochrome used (e.g. CD4-FITC).

☒ The axis scales are clearly visible. Include numbers along axes only for bottom left plot of group (a 'group' is an analysis of identical markers).

☒ All plots are contour plots with outliers or pseudocolor plots.

☒ A numerical value for number of cells or percentage (with statistics) is provided.

Methodology

Sample preparation	Cells from submandibular glands and primary tumors were isolated as detailed in the Methods.
Instrument	FACS Aria (BD Biosciences)
Software	FlowJo
Cell population abundance	Representative abundance of relevant cell populations are provided in the gating strategy (Supplementary Fig. 1a).
Gating strategy	The FSC/SSC gating strategy was used to eliminate cell debris, fragments and pyknotic cells with both low or high FSC/SSC reads. Subsetting gating strategy (FSC area and height) was used to identify the single-cell population and live cells further selected after exclusion of DAPI-positive cells.

☒ Tick this box to confirm that a figure exemplifying the gating strategy is provided in the Supplementary Information.
